# Depolymerization of Nylon‑6 over a Supported Ruthenium Catalyst to ε‑Caprolactam

**DOI:** 10.1021/acsenvironau.5c00272

**Published:** 2026-04-09

**Authors:** Prabin Dhakal, Abdenour Achour, Phuoc Hoang Ho, Aqsa Noreen, Derek Creaser, Louise Olsson

**Affiliations:** Chemical Engineering, Competence Centre for Catalysis, 11248 Chalmers University of Technology, Gothenburg 41296, Sweden

**Keywords:** depolymerization, nylon-6, ε-caprolactam, ruthenium, zirconia

## Abstract

Plastic recycling is a crucial process to establish a circular economy in the plastic industry and combat the environmental threat caused by plastic waste. Catalytic hydro-depolymerization, which typically uses molecular hydrogen and metal catalysts, is a promising approach to recycling plastic waste. Recent studies have mainly focused on polyolefin plastics, while heteroatom-containing plastics are also consumed in large quantities and are less studied. In this work, we present a novel heterogeneous catalytic process where nylon-6 is efficiently hydro-depolymerized to produce its monomer (ε-caprolactam). Using ruthenium supported on zirconia with activated hydrogen as an active and selective catalyst, we received 94% yield of ε-caprolactam, with only 3.4% hexamethylenimine as a byproduct, for the breakdown of nylon-6 at 350 °C and 30 bar H_2_. The depolymerization of nylon-6 to ε-caprolactam is shown to be sensitive to Ru particle size, with the disruption of the semicrystalline structure of nylon-6 being a prerequisite for C–N bond cleavage. A low metal loading of 2.3 wt % Ru, with a particle size of 9.5 ± 2.3 nm, was the most efficient catalyst. XPS and H_2_-TPR revealed that the smaller Ru particles were easier to reduce, and this is suggested to be the reason for the higher activity. Moreover, we propose that Ru-supported catalysts, in conjunction with activated hydrogen, exert a synergistic effect, facilitating both the alteration of crystallinity and the cleavage of C–N bonds, thereby enhancing the depolymerization rate of nylon-6. The robustness of the catalyst system is studied using plasticizers and additives. Interestingly, the addition of water could suppress byproduct formation, resulting in 100% selectivity. Additionally, the reusability of the catalyst is demonstrated.

## Graphical abstract

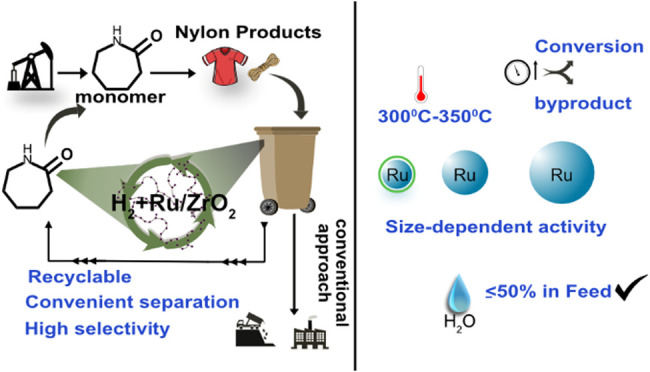

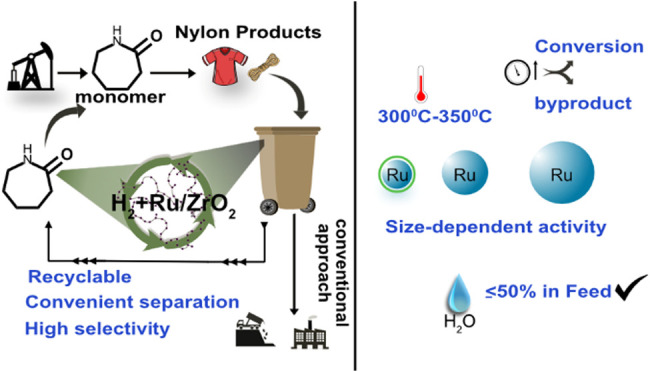

## 1 Introduction

Plastics are artificial polymers that contain repetitive units of organic molecules called monomers. Inexpensive, durable, chemically inert, easily shaped, and multifunctional are characteristics that have made plastics ubiquitous in our daily lives.^1^ Due to these properties, plastics have an advantage over other materials such as wood and metals, which has expanded the plastics economy throughout the globe. In Europe (EU27+ Norway, Switzerland, and the United Kingdom), approximately 50,000 plastic industries were operating, employing 1.5 million people with a turnover of 330 billion Euros in 2020.^2^ The production of plastics, in Europe, was around 55 million tons during 2020, while worldwide production of plastics was 367 million tons.^2^ Out of the various types of plastics, polyolefins like polyethylene (PE) and polypropylene (PP) account for about half of the market share, while other plastics with heteroatoms like polyamides (PA), polycarbonates (PC), polyethylene terephthalate (PET), and polyurethane (PU) make up more than 30% of the world’s plastic production.^2^ Additionally, plastic production has been growing at an annual rate of 8.4% since 1950, and the global production of plastics is expected to reach 1800 million tons by 2050.^3^ For instance, nylons, a class of polyamide, are projected to grow at an annual rate of 7.6% over the period of 2022–2030.^4^

Along with the increase in production of plastics, plastic pollution is also increasing. Annual generation of plastic waste is estimated to be 258 million tons, whereas the recycling rates of plastics are at around 14–18% at the global scale.^5^ Conventionally, a small fraction of plastic waste is processed using mechanical recycling–melting and extrusion of the plastic to form new products. This method of recycling is not an ideal option, as the number of repetitive cycles of melting and extrusion of plastics results in loss of strength and downgrading. The remaining plastic waste ends up in either landfills or incineration plants.^6^ Plastic waste is generated in large quantities and is not recycled, which means it can end up in the environment and cause a variety of environmental and health problems. Moreover, the plastics accumulated in the environment decompose over hundreds of years; hence, they fragment into micro- or nanoplastics and enter the food chain. This causes bioaccumulation of chemicals, causing several health-related issues, which is still under study.^5^ Thus, there is a need for new technologies to increase the recycling rate and avoid these issues.

Chemical upcycling of plastic waste has emerged as a substitute for the existing mechanical recycling process, in which waste plastic is depolymerized into monomers that can be used to make plastic again, to close the loop. Recently, research has focused on establishing depolymerization processes such as hydrolysis, ammonolysis, gasification, pyrolysis, methanolysis, photodegradation, hydrocracking, electrocatalysis, and hydrogenolysis as favorable approaches for plastic waste recycling.^7,8^ Unfortunately, most of these processes are not adopted commercially due to their energy intensity and the inseparability of impurities from products. Among the approaches, the concept of metal-catalyzed reductive depolymerization has shown promising results, providing higher selectivity for the desired products. In comparison to other conventional depolymerization techniques, catalytic hydro-depolymerization processes generate less stoichiometric waste, making them environmentally friendly and sustainable. Lately, there has been intensive research on the catalytic hydro-depolymerization of polyethylene (PP)^9,10^ and polypropylene (PE).^11,12^ Various types of metal catalysts have been investigated, yielding impressive results in breaking down long-chain polymers into short-chain hydrocarbons.^12–15^ However, catalytic depolymerization of nylons is among the least investigated cases, seemingly due to their resistance to chemicals and requirements for strong acid or base catalysts.

Nylon-6 (polyamide-6) is a widely used polyamide owing to its excellent properties, including high chemical resistance, good tensile strength, and abrasion resistance, combined with its low cost, which accounts for approximately two-thirds of the polyamide global market.^16^ These properties arise from the amide linkage (−C­(=O)–NH−), in which the distinct electronic characteristics of oxygen and nitrogen atoms stabilize its resonance.^17^ This electronic delocalization reduces the electrophilicity of the carbonyl carbon compared with other carboxylic acid derivatives, resulting in low reactivity toward nucleophilic attack.^18^ Nevertheless, catalytic reduction of amides can be achieved, although it typically requires relatively harsh conditions, at hydrogen pressures above 100 bar and temperatures near 200 °C. The reaction proceeds via cleavage of either C–O or C–N bond, with the latter pathway yielding alcohols or lactams.^19^ Among noble metal catalysts studied for amide hydrogenation, ruthenium (Ru) exhibits superior activity due to its strong hydrogen dissociation capability and moderate hydrogen adsorption strength. Effective overlap between the d-orbitals of Ru and σ* antibonding orbitals of hydrogen enables efficient hydrogen activation and generation of reactive surface H species.^20^ In addition, Ru preferentially adsorbs carbon heteroatom bonds, such as C–O, compared to Pt and Pd, while exhibiting a lower affinity for C–C bonds, which has led to its extensive use in CO_2_ hydrogenation, biomass hydrodeoxygenation, and H_2_ evolution reactions.^20–23^

Traditionally, homogeneous Ru catalysts have been well-documented in the reductive hydrogenation of amides. More recently, Kumar et al. illustrated the use of a Ru homogeneous catalyst for hydrogenative depolymerization of nylon-6 (Scheme 1a).^24^ They demonstrated carbon–nitrogen (C–N) cleavage in nylon-6 to form amino alcohols using dimethyl sulfoxide (DMSO), which presumably dissolves nylon at high temperatures. Similarly, Zhou et al. reported formation of diamines and diols through depolymerization of polyamides and polyurethane.^25^ Their strategic approach involved C–N cleavage using tetrahydrofuran (THF) and a homogeneous Ru catalyst. When employing nylon-6, neither of these approaches produced its industrial monomers, i.e., ε-caprolactam. Thus, the development of an efficient catalytic system for the depolymerization of nylons is highly desirable for achieving a closed-loop process. In this context, supported catalysts are preferred over homogeneous Ru systems in industrial applications due to their easier separation, higher thermal stability, recyclability, and tunable selectivity under demanding reaction conditions.^26^ ZrO_2_ has emerged as a versatile catalyst support owing to its well-documented thermal and mechanical stability, resistance to sintering under reaction conditions, and favorable metal support interactions that promote high metal dispersion.^27,28^ Recent application of ZrO_2_ was reported in a study by Tomita et al., which examined the hydrolysis of nylon and received a selectivity to ε-caprolactam of 84% with a yield of 81% using as much as 43% catalyst (ZrO_2_) compared to the nylon amount.^29^

**Scheme 1:**
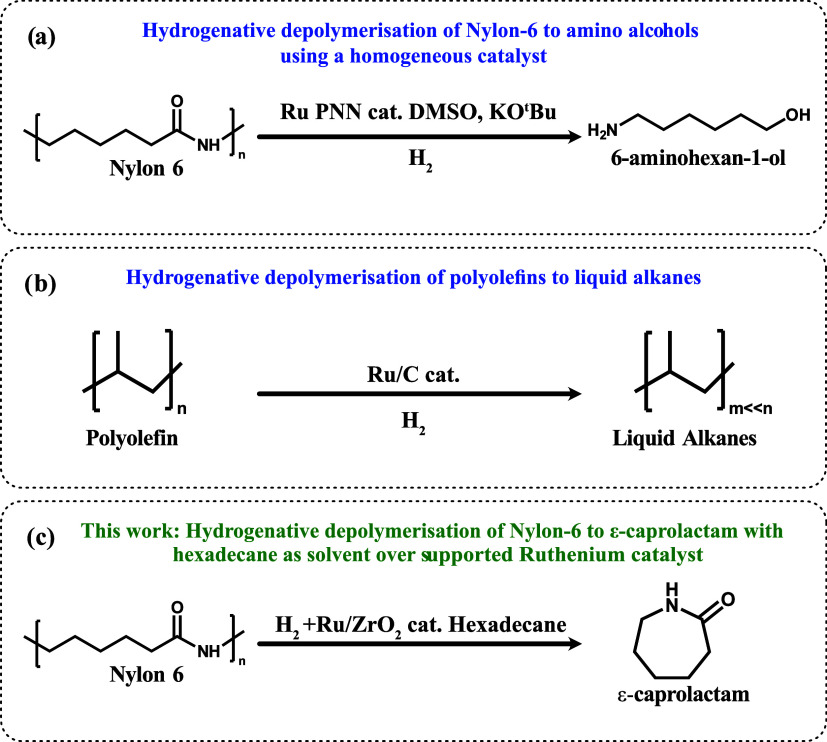
Examples of Ru-Based Catalysts for Polymer Depolymerization^a^

a (a) Hydrogenolysis of nylon-6 to amino alcohols using a Ru PNN homogeneous catalyst.^24^ (b) Hydrogenolysis of PE over a carbon-supported Ru catalyst into liquid alkanes.^10^ (c) This work: hydro-depolymerization of nylon-6 over Ru/ZrO_2_ to ε-caprolactam.

Some studies have shown the hydrogenation of primary and secondary amide linkages using different types of supported noble metal-based catalysts.^19,30–32^ In this direction, Tamura et al. showed the selective hydrogenation of amide linkages in various linear and cyclic amides to produce alcohols, amines, and other products using a supported Ru catalyst.^33^ Similarly, supported Ru catalysts have been studied for ammonolytic hydrogenation of polyamides to produce primary and secondary amines, diamines, and primary amides.^34^ However, their approach exhibited significant difficulties in converting PA6.6, resulting in only 36 wt % hexamethylenimine (Azapane) and unreacted plastic. Ru catalysts have recently been reported for various polymers as well. Rorrer et al. published their work on hydrogenative depolymerization of polypropylene and mixed polyolefins to light alkenes over Ru/C (Scheme 1b).^10^ Furthermore, Ru supported on ZrO_2_ has shown better activity with suppression of gas formation compared to other metal oxide supports such as CeO_2_, SiO_2_, TiO_2_, and Nb_2_O_5_ for depolymerization of polyethylene to produce liquid fuel (C5–C21).^35^

However, according to our knowledge, there is only one study available where a heterogeneous catalyst has been used to reductively depolymerize nylon-6, but in this study, the product was methane.^36^ Wu et al. received a selective hydrogenation using Ru/CeO_2_ of nylon-6 in solventless conditions at 325 °C with 84 and 96% yield of methane within 2 and 24 h, respectively.^36^ Our objective is to produce ε-caprolactam from depolymerization, since ε-caprolactam is the monomer in nylon production, and this could facilitate a closed-loop recycling. In this work, we demonstrate, for the first time according to our knowledge, an efficient and selective catalytic process for reductive depolymerization of nylon-6 to produce its monomer (ε-caprolactam) in the presence of a heterogeneous catalyst. For this reaction, we used Ru/ZrO_2_ as a catalyst, with n-hexadecane (n-C_16_) as a solvent, and the global reaction is depicted in Scheme 1c. We demonstrate that Ru/ZrO_2_ exhibits highly active catalytic properties and explore it under varying process parameters. Our approach requires conditions and a catalyst enabling successive C–N cleavage and cyclization for the transformation of nylon-6 to its industrial monomer, i.e., ε-caprolactam. We received a maximum of 94% yield of ε-caprolactam with a selectivity of 95% using only 5 wt % catalyst compared to the nylon amount. Thus, we used a factor of 9 times less catalyst (5 versus 43% catalyst) compared to the hydrolysis study by Tomita et al.,^29^ and we simultaneously received significantly higher selectivity (95 versus 84%). Additionally, we demonstrated a convenient separation of ε-caprolactam from the reaction mixture, since a heterogeneous catalyst was used. Moreover, with the addition of water to the process, a selectivity of 100% to caprolactam was found, with a yield of 84%. Here, we demonstrate that under the applied catalytic conditions, such a process can be selective and also energy-efficient for the upcycling of polyamide waste directly into usable monomers to build virgin polymers.

## 2 Methodology

### 2.1 Materials

The following chemicals were received and used as is, without further purification: ε-caprolactam (99% Sigma-Aldrich), hexamethylenimine (99% Sigma-Aldrich), dioctyl ether (99% Sigma-Aldrich), trifluoroacetic Anhydride (99% Sigma-Aldrich), and urea (>99% Sigma-Aldrich). Hexadecane (99% Sigma-Aldrich) and dimethyl sulfoxide (99%, VWR International) were used as solvents for different purposes. Nylon-6 pellets (2 mm, Sigma-Aldrich) were used as feedstock, with a viscosity-average molecular weight of 10,000 g/mol as per the supplier. Ruthenium­(III) nitrosyl nitrate solution in nitric acid (1.2 wt % ruthenium, Sigma-Aldrich) was used as the Ru precursor for catalyst preparation. Similarly, zirconium­(IV) oxynitrate hydrate (99% Sigma-Aldrich) was used as the source of zirconia.

### 2.2 Catalyst Preparation

Zirconia was prepared via a sol–gel method using urea as the precipitating agent. 30 g of urea was slowly added to a solution containing 17 g of zirconia in 250 mL of deionized water. The mixture was stirred continuously, heated to 90 °C, and refluxed for 18 h. The resulting precipitate was isolated by centrifugation and washed excessively with deionized water to produce a white solid, which was dried at 100 °C for 48 h.

The Ru catalyst was prepared by a wet impregnation method. First, the zirconia powder obtained was calcined at 500 °C for 5 h in air. Then, the calcined zirconia powder was dispersed in excess deionized water while stirring for 25 min. The predetermined volume of the ruthenium precursor was added dropwise, and the mixture was stirred at room temperature for 15 h. The slurry obtained was dried at 80 °C overnight to eliminate water and then calcined at 400 °C for 5 h with a heating ramp of 2 °C/min. Before activity tests, the catalyst was reduced at 250 °C in 10 bar of H_2_ for 2 h following a heating ramp of 10 °C/min. After cooling to room temperature, the catalyst was passivated in a stream of 25 N mL/min of 2% O_2_/Ar for 30 min at atmospheric pressure. A controlled passivation step forms a thin protective layer of oxide that allows safe handling and transfer of the highly reduced catalyst while preserving the metallic phase. Oxygen passivation after a reduction treatment of Ru- and other metal-based catalysts is commonly employed in the catalysis literature.^37,38^ to prevent uncontrolled and complete oxidation of the freshly reduced metallic phase upon exposure to air.

### 2.3 Catalyst Characterization

X-ray diffraction of all powdered catalyst samples was measured using a Bruker D8 powder diffractometer with Cu–Kα radiation (1.542 Å). Approximately 0.1 g of the calcined samples were mounted on the zero-background sample holder to obtain the data at 40 kV and 40 mA. The samples were scanned over the 2θ range of 10–70° with a step size of 0.04° to obtain the diffractogram. Elemental compositions (wt % of Ru) of the catalysts were analyzed using X-ray fluorescence (XRF) spectroscopy equipped with a Rh source operated at 60 kV and 125 mA. N_2_ physisorption was carried out using a Micrometrics Tristar II 3000 at −196 °C. Before the analysis, all the catalyst samples were degassed at 200 °C overnight in a flow of nitrogen gas. The surface area and pore volume were calculated using the Brunauer–Emmett–Teller (BET) theory and the BJH method, respectively. CO-chemisorption was performed to analyze the dispersion and crystal size of the metal using an ASAP 2020 plus (Micrometrics) instrument. Around 0.2 g of the catalyst was placed in a U-shaped reactor tube packed with quartz wool placed both up- and downstream. Prior to the analysis, the samples were degassed at 250 °C (heating rate of 10 °C/min) for 25 min in a flow of He and reduced under a flow of hydrogen for 2 h at 250 °C. Before the measurement was started, the sample was evacuated under vacuum. A pressure range of 80–600 mmHg was used for the CO adsorption measurement using a two-step isotherm method. The CO adsorption step was followed by evacuation for only 30 min to remove loosely physisorbed CO, followed by a second CO exposure step. The dispersion was determined as the difference between the two CO adsorption steps. The stoichiometry factor was determined from the CO–DRIFTS analysis by using the Beer–Lambert principle adaptation, where it was assumed that molar absorptivity is the same for all the species and the area is proportional to the quantity of CO molecules adsorbed.

In situ diffuse reflectance infrared Fourier transform spectroscopy (DRIFTS) spectra were measured using a Bruker Vertex 70 spectrometer fitted with an MCT detector and CO as the probe molecule. A spectral range of 3500–700 cm^–1^ was obtained using a 4 cm^–1^ resolution. Prior to analysis, the samples were reduced under a flow of 4% H_2_/Ar for 1 h at 250 °C. The residual H_2_ was flushed with Ar, and the spectrum was measured at 35 °C after exposure to 1000 ppm CO in Ar for 1 h.

H_2_-TPR (H_2_temperature-programmed reduction) was analyzed using a calorimeter (Setaram Sensys) equipped with mass flow controllers. Approximately 40 mg of the sample was placed in a quartz reactor tube. The samples were heated up to 300 °C in a flow of Ar to remove moisture prior to analysis. The reduction measurement was conducted under a constant flow of 3.5% H_2_ in Ar at 100 N mL/min. The sample was heated from 25 to 800 °C at a rate of 10 °C/min. The morphology of the catalyst was investigated using high-resolution transmission electron microscopy (HRTEM) with a FEI Titan 80–300 microscope. Reduced Ru samples were dispersed in ethanol and deposited over a 3 mm copper grid containing lacey carbon (Ted Pella, Inc.). Particle sizes were analyzed using Gatan Digital Micrograph software.

X-ray photoelectron spectroscopy (XPS) was performed on a PHI5000 VersaProbe III-scanning XPS microprobe with an Al–Kα monochromatic source to find the oxidation state of the catalyst. Catalyst powders were reduced in a batch reactor at 250 °C at 10 bar. The reactor was thereafter cooled to room temperature, and flushed several times with N_2_, which was followed by passivation with 2% O_2_ for 30 min before the XPS measurements were performed.

### 2.4 Activity Test

Prior to activity testing, nylon-6 samples (Sigma-Aldrich) were frozen with liquid nitrogen and then crushed using a grinder. The crushed powder was sieved to obtain particles less than 500 μm. The depolymerization reaction was carried out in a 450 mL Parr batch reactor. In a typical experiment, the reactor was filled with 3 g of nylon-6, 57 g of n-hexadecane (n-C_16_), and 0.15 g of catalyst. The reactor was then sealed, purged first with nitrogen and then hydrogen, brought to the desired gauge pressure, and heated to the desired temperature at 10 °C/min. Stirring was kept constant at 1000 rpm throughout all the experiments. After the predetermined reaction time, the reactor was cooled to room temperature by quenching in a water bath, and at room temperature, the reactor was flushed several times with N_2_. At the end of the experiment, the reactor contained an n-C_16_ soluble phase, along with n-C_16_ insoluble solid polymers. The crude mixture (n-C_16_ phase) was separated using vacuum filtration. Thereafter, the reactor was washed with acetone to collect the n-C_16_ insoluble product (acetone phase), and acetone was evaporated in an airflow to recover it. The solid fraction referred to as total solid was recovered by heating the reactor to 180 °C for a few minutes, and then the partly molten polymer was transferred to a glass vial for analysis.

### 2.5 Product Analysis

The n-C_16_ phase and acetone phase were analyzed and quantified by gas chromatography coupled with mass spectrometry (GC-MS). Approximately 0.2 g of the product recovered from the acetone phase was diluted with DMSO to make a volume of 10 mL, and 100 μL of dioctyl ether was added as an internal standard. Similarly, the n-C_16_ phase was mixed with 100 μL of dioctyl ether to make a volume of 10 mL. The analysis was performed with an Agilent 7890 B GC fitted with a VF-1701 MS column (30 m × 0.25 m × 0.5 μm), coupled with a 5977A MSD mass spectrometer. The column flow was set to 2 N mL/min, and the injector temperature was set to 280 °C. The oven was programmed initially at 50 °C, held for 5 min, and then heated to 280 °C at a rate of 20 °C/min. ε-Caprolactam and hexamethylenimine were used as external standards for calibration. The monomer yield was calculated using eq 1.

1 $$\mathrm{yield}=\frac{\mathrm{mass}\;\mathrm{of}\;\mathrm{product}\;\mathrm{monomers}}{\mathrm{mass}\;\mathrm{of}\;\mathrm{feed}\;\mathrm{nylon}\;\left(\mathrm{mi}\right)}\times 100\%$$

2 $$\mathrm{selectivity}=\frac{\mathrm{amount}\;\mathrm{of}\;\mathrm{each}\;\mathrm{product}}{\sum \mathrm{amount}\;\mathrm{of}\;\mathrm{each}\;\mathrm{product}}\times 100\%$$

The identity of the product was confirmed using nuclear magnetic resonance (NMR) spectroscopy with 1,4-dinitrobenzene as the standard.

The mass of the total solid remaining after the reaction was used to determine the conversion of nylon-6. Approximately 0.2 g of the total solid obtained was suspended in 5 mL of dichloromethane, and 100 μL of trifluoracetic acid anhydrous (TFAA) was added and stirred for 18 h. Unreacted nylon-6 was completely soluble, which was removed through filtration. Conversion of nylon-6 was calculated using eq 3.

3 $$\begin{array}{l} \mathrm{unconverted}\;\mathrm{polymer}=\mathrm{mass}\;\mathrm{of}\;\mathrm{total}\;\mathrm{solid}-\mathrm{mass}\;\mathrm{of}\;\mathrm{total}\;\mathrm{solid}\;\mathrm{insoluble}\;\mathrm{in}\;\mathrm{TFAA}-\mathrm{mass}\;\mathrm{of}\;\mathrm{catalyst}\\ \mathrm{conversion}\;\left(\%\right)=\frac{\mathrm{polymer}\;\mathrm{in}\;\mathrm{feed}-\mathrm{unconverted}\;\mathrm{polymer}}{\mathrm{polymer}\;\mathrm{in}\;\mathrm{feed}}\times 100 \end{array}$$

The total solids were further analyzed using differential scanning calorimetry (DSC) and elemental analysis to monitor the variation of unreacted nylon from the feed nylon. The melting point of the total solids was determined using a DSC2 (Mettler Toledo). During the analysis, less than 2 mg of the sample was heated from 35 to 350 °C at 10 °C/min and was purged with a constant nitrogen flow of 50 N mL/min. The melting point temperature and full width at half-maximum (fwhm) were evaluated by normalizing the curve with the sample mass. The elemental analysis, providing the content of nitrogen, sulfur, hydrogen, and carbon, was measured using an Elementar vario Macro cube. Thermal gravimetric analysis (TGA) was performed using a TGA DSC3+ (Mettler Toledo), where the sample was heated in a constant N_2_ flow of 50 N mL/min in a 70 μL alumina crucible.

### 2.6 Catalyst Reusability Test

To regenerate activity and test for reusability, 2.3 Ru/ZrO_2_ was first used in a regular run at 350 °C for 2 h with a polymer-to-catalyst mass ratio of 20:1 (fresh catalyst). After the reaction, the catalyst with total solid was collected after filtration, washed with acetone, and dried at room temperature overnight. The total solid from 3 different batches conducted under the same reaction conditions was collected and calcined at 400 °C for 5 h. Next, the recovered powdered catalyst was mixed with 20 wt % fresh catalyst to make up for losses during filtering and collection for each run. The mixture of the catalyst was then reduced and tested for its depolymerization activity following the protocol in Section 2.4. N_2_ physisorption and TGA were also used for the investigation of the regenerated catalyst.

## 3 Results and Discussion

### 3.1 Catalyst Characterization

To gain a better understanding of the catalyst performance, the support and freshly prepared catalyst were characterized by XRF, N_2_ physisorption, XRD, TEM, CO adsorption, XPS, and DRIFTS. Table 1 shows the summary of the characterization results of the different catalysts. Several batches of the zirconia support were prepared using the sol–gel method and impregnated with different loadings of Ru metal, as shown in Table 1. The Ru loadings measured by XRF were close to the nominal values. The N_2_ physisorption measurements showed that the synthesized zirconia exhibited an average specific surface area of 69.2 m^2^/g with a standard deviation between different synthesized batches staying within 5%. Table 1 shows the N_2_ physisorption data for two different batches prepared over a period of a month, showing the reproducibility of the synthesis process. The average pore diameter of the zirconia was 3.2 nm with an average pore volume of 0.077 cm^3^/g STP. Furthermore, the specific surface area decreased significantly with the Ru loading, and a decrease in pore volumes followed a similar trend. These results are expected, since higher loading results in more blockage of the pores of the support. The XRD spectra for the as-prepared catalysts are illustrated in Figure S1a (Supporting Information (SI)). The zirconia exhibits three polymorphs: monoclinic, tetragonal, and cubic. The as-prepared zirconia illustrated tetragonal peaks accompanied by smaller monoclinic peaks. The peaks observed for 2θ values at 29–30.6, 33.5–36, 48.52.5, 58–61.5, and 64.5–62 are attributed to the tetragonal phase, and the peaks at 27–29 and 30.6–32.2 are attributed to the monoclinic phase and are in good agreement with the reported data (PDF 04–013–6616 and PDF 04–013–6620). The average crystallite size for the tetragonal phase was 15.5 nm, and for the monoclinic phase, it was 10 nm. However, after Ru impregnation, the spectra due to the Ru metal are weak and overlapped with signals from the ZrO_2_ support. Hence, to estimate the metal particle size correctly, the pattern obtained by subtracting the ZrO_2_ support spectra from that of Ru/ZrO_2_ was used, as shown in Figure S1b (SI). The features with low intensity observed for 2θ values of 41, 58, 62, and 67 correspond to (200), (220), (112), (302), and (202) crystal planes, respectively, showing the low crystalline state of the supported RuO_2_ for these planes. However, the peaks observed at 2θ values of 28, 32, and 55 represent (110), (101), and (211) planes, respectively, and indicate the highly crystalline state of RuO_2_.^39^

**Table 1:** Summary of Characterization Results of Catalysts Using ICP, H_2_-TPR, N_2_-Physisorption, CO-Chemisorption, XRD, and TEM

		**XRF**	**H**_**2**_ **-TPR**	**N**_**2**_ **physisorption**		**CO-chemisorption**	**average particle size** (nm)		
**catalyst**	**nominal metal loading** (wt %)	**metal loading** (wt %)	**degree of reduction** (%)	**specific surface area** (m^**2**^ **/g)**	**pore volume** (cm^**3**^ **/g)**	**dispersion** (%)	**XRD**	**chemisorption**	**TEM**
ZrO_2_				69.6	0.077				
ZrO_2_				69.9	0.069				
2.3 Ru/ZrO_2_	2	2.3	19.0	59.0	0.072	14.1	11.7	9.5	9.5
3.5 Ru/ZrO_2_	4	3.5	18.6	32.9	0.070	11.2	14.1	11.9	13.9
6.9 Ru/ZrO_2_	8	6.9	14.5	24.4	0.063	7.4	14.6	18.1	17.8

The presence of Ru oxide, as indicated by XRD, suggests that Ru could be reduced to its metallic state via pretreatment prior to the depolymerization test. Therefore, it was necessary to identify the reduction temperature and reducibility of the catalyst. The reducibility was investigated by H_2_-TPR, and the results are shown in Figure S2 (SI). From the results, the reduction peak is observed between 132 and 150 °C, ensuring that the Ru species at all loadings were reduced during the applied pretreatment at 250 °C prior to the activity tests. The early reduction can be attributed to the conversion of particles of RuO_*x*_ or RuO_2_ to metallic Ru. Higher temperature reduction peaks (not seen in this case) are generally associated with the reduction of ruthenium oxides that have a strong interaction with the ZrO_2_ support. A broad peak below 200 °C, as reported in the literature, is generally associated with the reduction of weakly bound Ru particles on the support.^40^ Additionally, the reduction peak for 2.3 Ru/ZrO_2_ is ∼18 °C lower compared to that of the 6.9 Ru/ZrO_2_ sample. This can be expected to cause an enhancement of the surface activity with the smaller Ru particles of 2.3 Ru/ZrO_2_. The degree of reducibility, calculated based on the assumption that the reducible species present on the catalyst are RuO_2_, is shown in Table 1 (details in Table S1, SI). A low degree of reducibility indicates the presence of a lower number of reducible species of Ru on the surface, and the degree of reducibility decreases with an increase in loading. Notably, Ru particles may sometimes remain partially unreduced even at low loadings. Lizandra et al. reported a similar trend when the metal loading on ZrO_2_ was 1 wt % and suggested that it was due to the presence of Ru species with lower reducibility.^41^

Besides reducibility, catalysts were analyzed by FTIR using CO as the probe molecule at 35 °C. The FTIR spectra of CO-absorbed on the reduced Ru/ZrO_2_ catalysts are shown in Figure S3 (SI). This technique is characterized by the fact that only exposed sites can be explored; subsurface or buried sites have no influence. Consequently, it gives information needed to understand the accessible or “working sites” involved in the catalytic reactions. The feature at 1637 cm^–1^ is associated with CO adsorption on zirconia. A broad band near 1995 cm^–1^ could be attributed to bridge-bound CO species absorbed on reduced Ru sites.^42^ A band near 2020 cm^–1^ could be assigned to linear-carbonyl species.^43^ The peak at 2065 cm^–1^ can be attributed to dicarbonyl species.^44^ The bands between 2120 and 2200 cm^–1^ are assigned to multicarbonyl species on Ru sites.^43^ These are common peaks seen in all three catalysts. It has been reported by Wang et al. that the use of a conventional CO/Ru stoichiometric factor of 1 provides an inaccurate dispersion of Ru sites.^11^ In addition, the use of a conventional pulse H_2_ method only accounts for metallic Ru, underestimating the dispersibility of Ru. Therefore, using these peak assignments from CO-DRIFTS results, the stoichiometric ratio was calculated (Table S2, SI), revealing a stoichiometric factor of 1.3 between CO and Ru.^43^

The Ru dispersion was measured with CO-chemisorption at 35 °C. As expected, the Ru dispersion decreased significantly with increasing metal loading. The total surface Ru estimated using CO-chemisorption only increased 1.6 times, even though the Ru metal loading increased by 3-fold from 2.3 to 6.9 wt % (Table S3, SI). Ru crystallite sizes estimated from the dispersion as hemispherical particles increased from 9.5 to 18.1 nm with an increase in the loading from 2.3 to 6.9 wt %.

The impact of metal loading on particle size was also analyzed using transmission electron microscopy (TEM). Figure 1 presents the high-resolution TEM (HRTEM) images along with particle size histograms of the reduced catalyst. Notably, distinct lattice fringes were observed, and varying interplanar distances were measured. Two grid stripes with interplanar spacings of 0.31 and 0.29 nm corresponding to the monoclinic and tetragonal phases of zirconia, respectively, were observed. These edges, as shown in the figure, were assigned to the (−111) planes of monoclinic zirconia and the (101) planes of tetragonal zirconia. On the other hand, the interplanar spacings of (110) and (101) planes of RuO_2_ particles are 0.31 and 0.25 nm, respectively. Despite the significant differences in *d*-spacing between the two zirconia phases, there was no notable difference between the *d*-spacing of the (−111) planes of zirconia and the (110) planes of RuO_2_ particles. However, the (101) RuO_2_ planes, with a *d*-spacing of 0.25 nm, were identified, and the particle size distribution was measured. As anticipated, the particle size increased with higher metal loading. The average particle size for 2.3 Ru/ZrO_2_ was 9.5 nm, increasing to 17.8 nm for 6.9 Ru/ZrO_2_. Ruthenium particles exhibited no preferential distribution between the monoclinic and tetragonal phases of zirconia.

**Figure 1:**
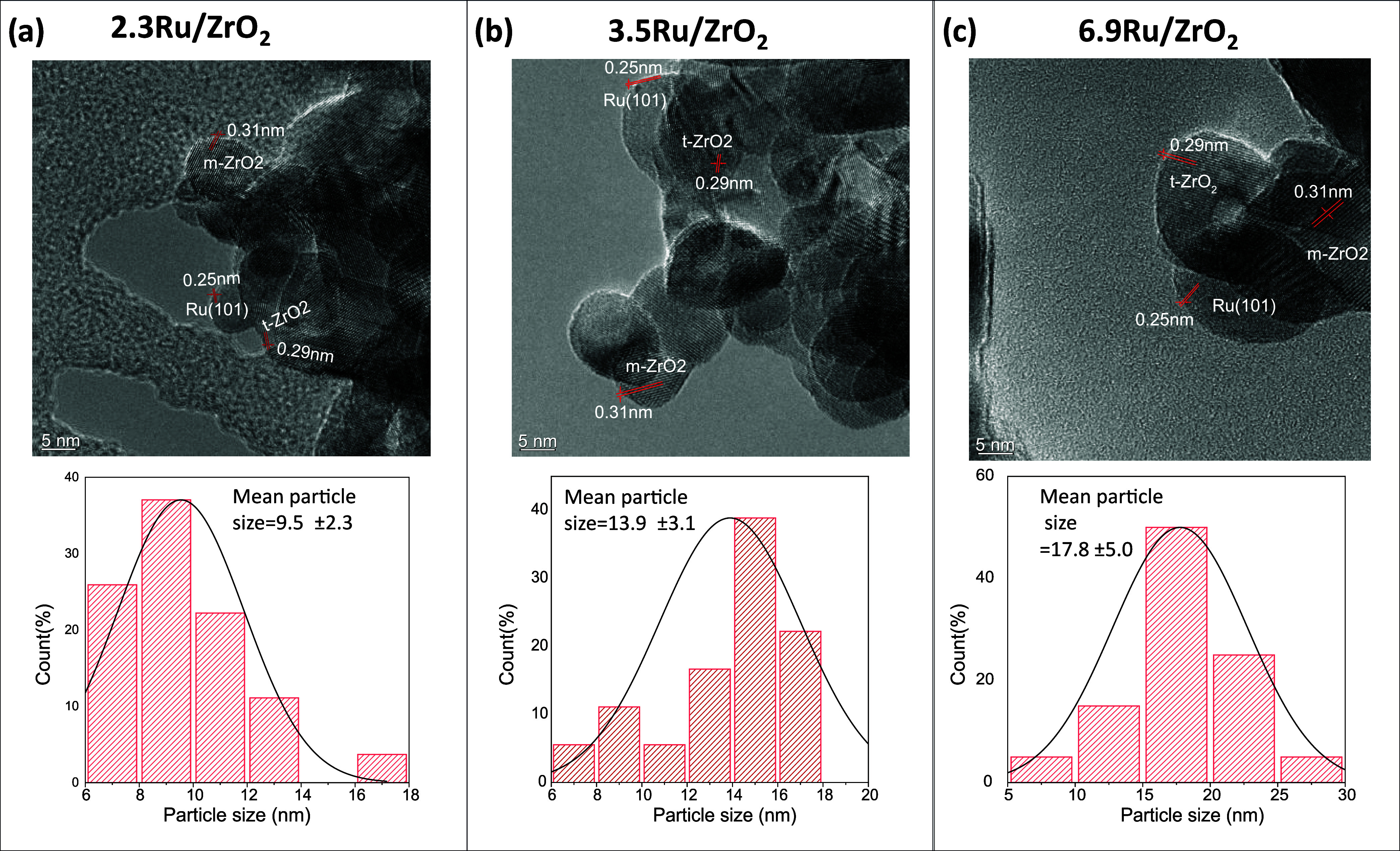
HRTEM images and histogram of (a) 2.3 Ru/ZrO_2_, (b) 3.5 Ru/ZrO_2_, and (c) 6.9 Ru/ZrO_2_.

To examine the influence of loading on the Ru oxidation state, XPS analysis was performed on the catalyst samples. Figure 2 shows the Ru 3d XPS profiles, for the as-prepared 2.3 Ru/ZrO_2_ sample and the reduced 2.3 Ru/ZrO_2_, 3.5 Ru/ZrO_2_, and 6.9 Ru/ZrO_2_. Given the complexity of the spectrum in this region due to the overlap of the C 1s peak and the Ru doublet (Ru 3d_5/2_ and Ru 3d_3/2_), deconvolution of the spectrum was necessary.

**Figure 2:**
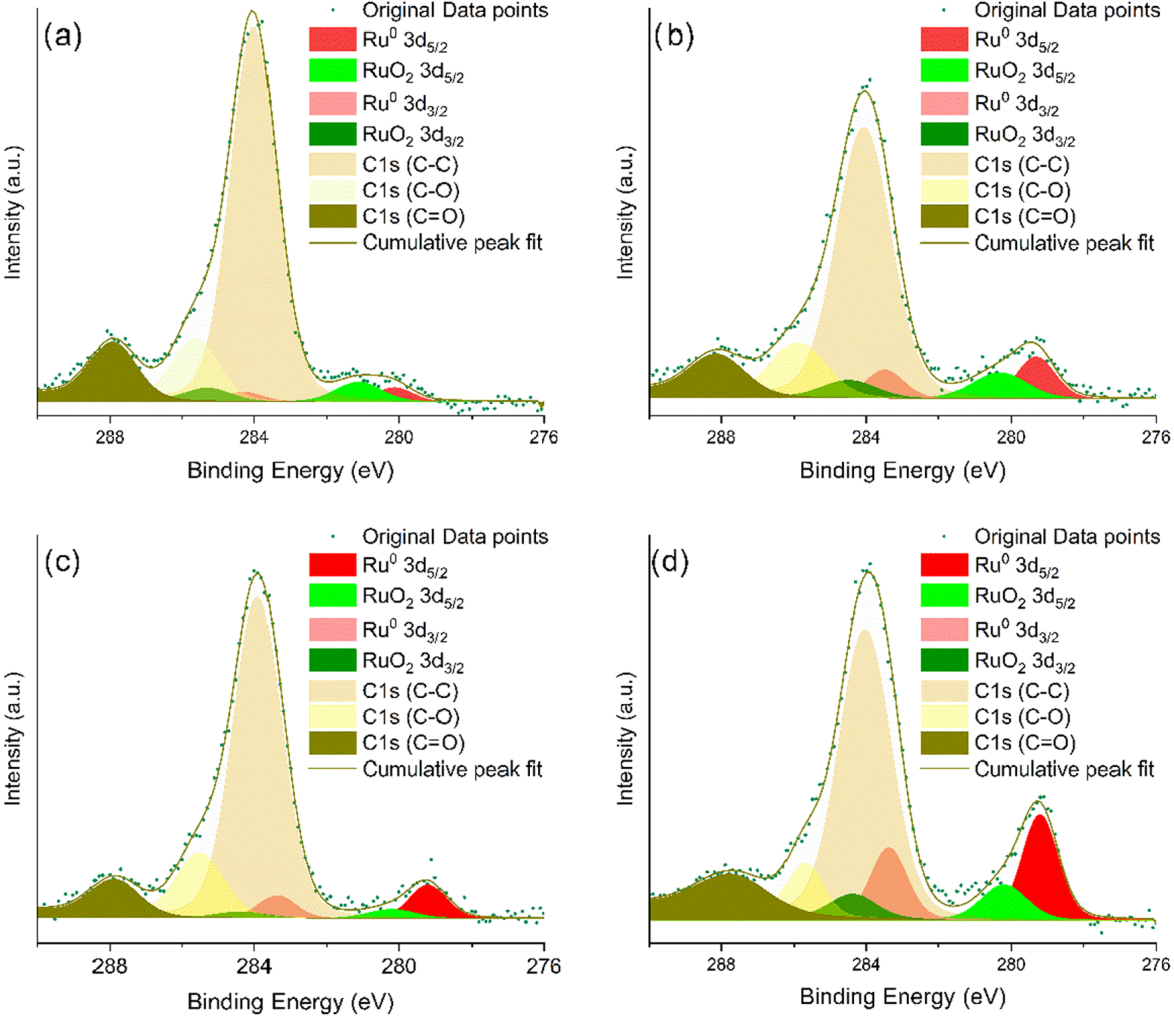
High-resolution XPS Ru 3d resolved spectrum of (a) as-prepared 2.3 Ru/ZrO_2_, (b) reduced 2.3 Ru/ZrO_2_, (c) reduced 3.5 Ru/ZrO_2_, and (d) reduced 6.9 Ru/ZrO_2_.

The Ru 3d peaks were fitted with states corresponding to metallic Ru^0^ at 279.5 eV and RuO_2_ at 280.7 eV. The Ru 3d_5/2_ region contains two Ru peaks representing Ru^0^ and RuO_2_, while the Ru 3d_3/2_ peaks are fitted with three Ru peaks, maintaining a constant separation between Ru 3d_5/2_ and Ru 3d_3/2_ of 4.1 ± 0.1 eV. The peak for C 1s is set at 284.8 eV to fully account for the region.

Analysis of the spectra indicates that Ru remains predominantly oxidized (Ru^4+^) before reduction. However, after reduction and passivation, the fraction of metallic Ru increases. For example, in 2.3 Ru/ZrO_2_, the metallic fraction rises from around 35 to 50% (Δ∼15%). These results are consistent with the H_2_-TPR, where only ca. 15–20% of the ruthenium was reduced (Table S1, SI). Peak fitting of the Ru 3d/C 1s region and the comparison between as-prepared (Figure 2a) and reduced and passivated (Figure 2b) 2.3 Ru/ZrO_2_ confirm that Ru^4+^ contribution diminishes after the reduction, while metallic Ru grows, accompanied by a slight shift in binding energy. These findings suggest that a portion of Ru is already present in the metallic state in the as-prepared catalysts and that this fraction increases with Ru loading. Consequently, the apparent reducibility derived from H_2_-TPR is lower, since part of the Ru does not require further reduction. The coexistence of metallic Ru^0^ and Ru^4+^ detected by XPS, along with the relatively low hydrogen consumption in TPR, is consistent with a core–shell structure, in which Ru nanoparticles comprise a metallic Ru^0^ core covered by a thin RuO_2_ shell. In this model, the surface-sensitive XPS technique preferentially detects the oxidized shell in as-prepared samples, whereas the metallic core contributes less to the measured surface signal. Reduction diminishes the shell contribution and exposes more metallic Ru, explaining the observed spectral changes.

In conclusion, catalyst characterization revealed the physicochemical characteristics of the catalysts with varying metal loadings. The following sections will address how these attributes affect catalytic activity.

### 3.2 Depolymerization Results

As a starting point, the depolymerization of nylon-6 was carried out at three different temperatures (250, 300, and 350 °C). The reaction conditions were 30 bar H_2_ pressure at room temperature, a reaction time of 5 h, and a polymer-to-catalyst mass ratio of 20. Figure 3a compares the product yield fractions (product masses from initial nylon-6 mass) for these temperatures for the catalyzed and uncatalyzed (blank) reaction cases. The catalyzed reactions were carried out with 2.3 wt % ruthenium on zirconium oxide (2.3 Ru/ZrO_2_) as the catalyst. To determine the actual conversion of nylon-6, it was necessary to characterize the total solids obtained after the reaction. The conversion of nylon-6 was assessed through a polyamide functionalization method based on trifluoroacetylation of its NH group.^45^ (see the SI for details).

**Figure 3:**
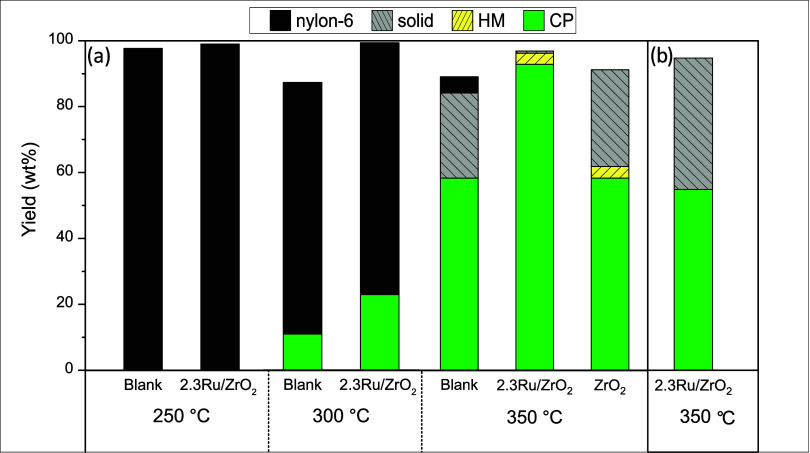
Nylon-6 depolymerization at varying temperatures, different gas atmospheres, and the presence of a catalyst. Reaction conditions: 57 g of hexadecane solvent; 3 g of nylon-6; polymer-to-catalyst mass ratio of 20:1; gas pressure of 30 bar; 5 h reaction time; “blank” indicates the absence of the catalyst. Gas atmospheres of (a) H_2_ and (b) Ar; CP: ε-caprolactam; HM: hexamethylenimine.

Initially, the depolymerization experiment was attempted at 250 °C, close to the melting point of nylon-6. The first peak at 226.3 °C, as measured by DSC (Table S5, SI), shows the endothermic change, corresponding to the melting of semicrystalline unreacted nylon-6.^46^ This melting point temperature ensured that the experimental temperature was higher than the melting point of nylon-6, and the reaction occurs with the polymer in a molten state. The TGA analysis showed that nylon-6 decomposes above 380 °C (Figure S4, SI), and the maximum experimental temperature was therefore limited to 350 °C to avoid self-degradation of the polymer. The effectiveness of the depolymerization activities was scrutinized by measuring the product distribution, elemental composition, and TGA/DSC of products. The measured total yields (see Figure 3) was between 88 to 99%, and the deviations from 100% yield are mainly due to losses during the different steps of product recovery. It is apparent from Figure 3 that no depolymerization occurred at 250 °C for both catalyzed and uncatalyzed reactions. The lack of activity just above the melting point could be due to the fact that the activation energy is too high for conversion at this low temperature. Consequently, higher temperatures were examined to overcome this.

Further elevating the temperature to 300 °C, the catalyst activity improved and resulted in a mixture of a crystalline product and a solid polymer at the end of the experiment, suggesting partial conversion of nylon-6. The crystalline product was collected as explained in Section 2.4. Indeed, the analysis with GC-MS revealed the formation of a 7-membered amide ring structure, i.e., ε-caprolactam (CP), with 10 and 23 wt % yield for the uncatalyzed (blank) and catalyzed reactions, respectively, as shown in Figure 3. The structure of the CP was further verified using proton and HSQC NMR (Figures S5 and S6, SI). Unfortunately, after 5 h of reaction at 300 °C, at least 76 wt % polymers were still intact following both the catalyzed and uncatalyzed reactions. Gratifyingly, when the experiment was performed with an increased temperature of 350 °C, there was a significant increase in the depolymerization activity, and with the catalyst, it led to almost quantitative conversion. The liquid products obtained from the catalyzed depolymerization contained 94 wt % CP and 3.4 wt % hexamethylenimine (HM). The uncatalyzed reaction at 350 °C resulted in 59.6 wt % CP without any yield of HM and with large solid byproduct formation. Interestingly, at 350 °C, the CP yield was similar for the blank experiment with H_2_ (Figure 3a) and the catalytic reaction with H_2_ replaced by Ar (Figure 3b). Similarly, when bare zirconia was used with H_2_, the CP yield was similar to that of the uncatalyzed reaction with H_2_, and also in this case, large solid byproduct formation was observed. Evidently, the depolymerization is highly efficient in the combined presence of both H_2_ and the active metal sites, even though the formation of CP does not consume H_2_. In addition to this, the initial and final reactor pressures at room temperature were found to be the same (30 ± 1 bar gauge pressure), indicating no significant consumption of H_2_ occurred. Apparently, the Ru metal sites and H_2_ together acted as the catalyst.

The products reported so far in the literature from hydrogenative depolymerization of nylon-6 were mainly amino alcohols, diols, and diamines using a homogeneous catalyst and alkanes and ammonia using a heterogeneous Ru/CeO_2_ and Pt/CeO_2_ catalyst in solvent-free conditions.^24,25^ Comparison of the GC results (area %) from reactions conducted with and without nylon-6 (Table S6, SI) confirmed that no significant contribution of hydrocarbons originated from nylon-6. Our suggested process is selectively forming ε-caprolactam, which is a large benefit, since ε-caprolactam is the monomer when producing nylon-6. Thus, we effectively convert nylon-6 back to its monomers. The long reaction times (24 to 72 h) used in the studies in literature using homogeneous catalysts^24,25^ could result from the high solubility of the polyamide in the polar solvents used, like DMSO. This is most likely caused by the interaction of the solvent with nylon-6, through hydrogen bonding, and explains why polyamides can be soluble in such solvents. Here, a nonpolar solvent is used, n-C_16_, which reduced the interaction between the polyamide and the solvent and resulted in a significantly faster reaction. The effects of polarity were also reported for cyclopentyl-methyl-ether during ammonolytic hydrogenation of polyamide-11 and polyamide-12, which resulted in low conversion at 200 °C and 16 h of reaction time.^34^ In another study, the use of 1,4-dioxane solvent resulted in lower conversion of nylon-6 at 150 °C compared to DMSO due to the solubility of the polymer.^24^ Furthermore, a study by Sadeghmoghaddam et al. has shown that the use of a polar solvent favors reactions involving hydrogenation of allyl alcohol to 1-propanol, whereas the use of a nonpolar solvent results in isomerization to propanal.^47^ This could also be partly due to the stronger interaction of polymer–catalyst in the presence of a nonpolar solvent, in contrast to a polar solvent that could strongly interact with the catalyst through its functional groups, thus inhibiting polymer–catalyst interactions. These factors may explain why n-C16 appears to be a favorable solvent for achieving high CP yield.

To summarize, in the literature, less than 2 wt % yield of caprolactam was previously reported, which is in a study where hydrogenolysis of a polyamide was performed using a homogeneous ruthenium PNN catalyst and DMSO as a solvent.^24^ However, in our process using a heterogeneous catalyst and a nonpolar solvent, we were able to retrieve 94% yield of ε-caprolactam (CP).

CP is an important industrial monomer, produced mostly using the Beckmann rearrangement, and used for the production of polyamides.^48^ Literature shows polar solvents with higher solubility toward the CP and that the solubility of CP decreases with an increase in chain length (C_6_–C_16_) and increases with the presence of polar active groups such as an ether, ketone, or alcohol.^49^ The poor mutual solubility of CP and the n-C_16_ solvent made the separation of CP easier from the reaction mixture due to its polarity, which is another advantage with this solvent. The easy separation could also be attributed to the melting point (66 °C, Figure S7, SI) of the CP, where at room temperature, a large amount of CP remained as insoluble crystals in n-C_16_, and therefore, CP was easily collected from the reactor by dissolving in acetone. However, a small amount of CP was still detected in the n-C_16_ phase (Table S7, SI). HM, on the other hand, is liquid at room temperature and has a low melting point (−37 °C). It was only found in the n-C_16_ phase, indicating that it is soluble in hexadecane at low concentrations.

In the presence of supported Ru and H_2_, the minimal solid yield was observed at 350 °C, indicating that Ru and H_2_ are crucial in facilitating the depolymerization of nylon-6 to CP. Later, during the investigation of total solids with respect to reaction time, it will be established that the formation of solids is probably because of prolonged exposure of nylon-6 to high temperatures. The catalyst along with H_2_ likely operates to accelerate the depolymerization process, thereby enhancing the conversion of the polymer into CP. Moreover, the Ru supported on zirconia with activated H_2_ working as the catalyst plays a significant role in determining the selectivity between the formation of solids and CP.

To better understand the role of the catalyst in the depolymerization, Ru/ZrO_2_ materials were synthesized containing Ru with loadings of 2.3 wt % (2.3 Ru/ZrO_2_), 3.5 wt % (3.5 Ru/ZrO_2_), and 6.9 wt % (6.9 Ru/ZrO_2_) as measured by XRF. The activity of the catalyst can be influenced by the metal support interaction.^50^ Thus, all structural properties of the support and the fabrication method of the Ru/ZrO_2_ catalysts were equivalent to ensure that the activity changes reflect the influence of changes in the properties of the metal particles. The uniform structural properties of the support were ensured by mixing the different batches of synthesized zirconia before impregnation with the metal precursor. The catalysts were subjected to the depolymerization of nylon-6 at a reduced experiment time of 2 h and at 350 °C, whereas all other parameters were identical to conditions for experiments in Figure 3.

Under the reaction conditions used, the yield of various products from the experiments are shown in Figure 4a. The yield of CP follows the order: 2.3 Ru/ZrO_2_ > 3.5 Ru/ZrO_2_ > 6.9 Ru/ZrO_2_. The depolymerization to CP increased in the presence of a catalyst compared to the uncatalyzed reaction (blank). The reaction produced a maximum yield of monomers over the 2.3 Ru/ZrO_2_ catalyst. Compared to the CP yield over 2.3 Ru/ZrO_2_, the yield decreased by 10 and 17 wt % over 3.5 Ru/ZrO_2_ and 6.9 Ru/ZrO_2_, respectively. To confirm the reproducibility of the depolymerization experiments, the experiment with 2.3 Ru/ZrO_2_ was repeated two more times. The average CP yield was found to be 82.2 ± 4.5 wt %, with a selectivity of 98 ± 4.5 wt % (Figure S9 and Table S9, SI).

**Figure 4:**
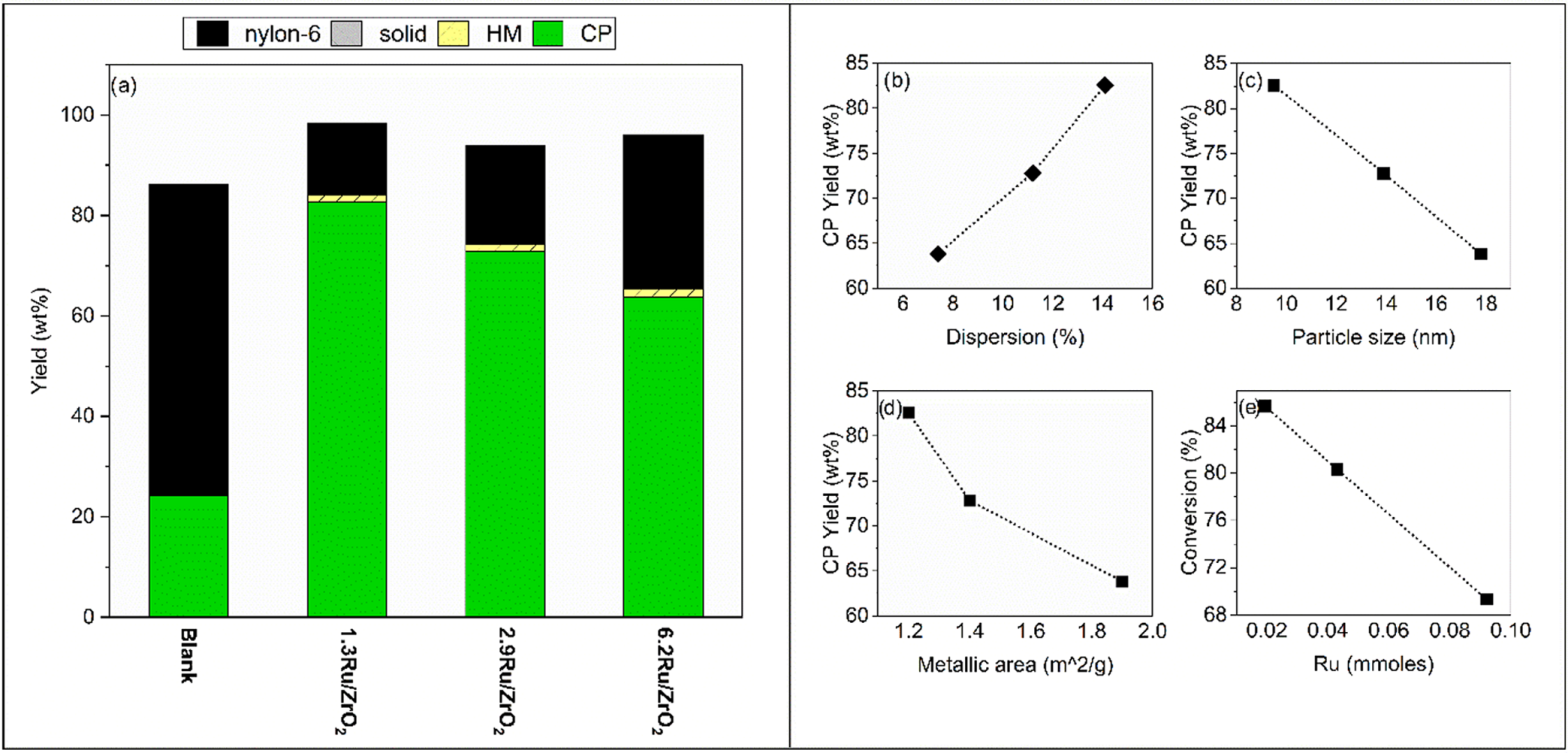
Nylon-6 depolymerization over Ru/ZrO_2_ catalysts with varying Ru loadings (a) and product yields vs catalysts. (b) CP yield vs Ru dispersion, (c) CP yield vs Ru particle size, (d) CP yield vs surface Ru moles determined by chemisorption, and (e) nylon-6 conversion vs Ru loading. Reaction conditions: solvent = 57 g of hexadecane; feed = 3 g of nylon-6; polymer-to-catalyst mass ratio = 20:1; temperature = 350 °C; pressure = 30 bar H_2_; reaction time: 2 h. CP = ε-caprolactam; HM = hexamethylenimine.

There are several indications in the literature that the performance of the catalyst can be closely related to the electronic properties of the catalyst and the metal particle size.^50,51^ Ru is a metal with high vacancies in its d-orbitals and a small atomic radius and therefore shows high activity through faster generation of electronic density, which helps to adsorb hydrogen and polymer. The performance of the catalysts is compared to the results of the characterization studies (Figure 4b–e). The 2.3 Ru/ZrO_2_ catalyst contains smaller particles as determined by HRTEM, which may contribute to enhanced depolymerization of the polymer.

The adsorption of polymers onto metal surfaces is a critical step in depolymerization processes, particularly for the depolymerization of nylon-6 to CP. Nylon-6 can interact with metallic Ru, Ru^4+^, and oxophilic zirconia through its functional groups, specifically C = O and N–H, forming various catalyst–polymer conformations.^24,29^ Thus, the depolymerization of nylon-6 could be significantly influenced by metal nanoparticles. However, it remains unclear whether the depolymerization reaction occurs on metallic Ru sites or Ru–support interfacial sites. The partial double-bond character of the nitrogen atom introduces steric hindrance to the formation of catalyst–polymer conformations, which could be exacerbated by larger metal particle sizes, hindering effective adsorption and favorable formation of polymer–catalyst conformations (Scheme 2). Additionally, the density of active sites at the metal support interface decreases with an increase in the particle size as the surface-to-volume ratio diminishes. Strong metal–support interactions, including charge transfer arising from differences in the Fermi levels of Ru and the zirconia support, are expected to occur predominantly at these interfacial regions.^52^ Such electronic interactions are more pronounced for smaller Ru particles, where a larger fraction of Ru atoms is located at or near the interface.^53^ Consequently, the enhanced depolymerization performance observed for smaller Ru particles can be the result of a combined effect of improved polymer adsorption, higher interfacial site density, and stronger metal–support interactions.

**Scheme 2:**
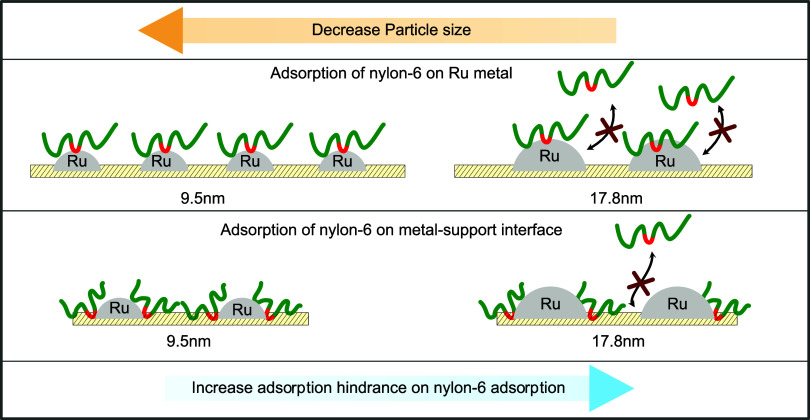
Illustration of the Particle Size Effect on the Adsorption Behavior of Nylon-6 on the Ru Metal and Ru–ZrO_2_ Interface

Despite similar types of adsorption sites across the three prepared catalysts, as indicated by CO-DRIFTS results, the impact of Ru particle size on nylon-6 depolymerization suggests that the process may be structure-sensitive. The formation of CP clearly decreases with increasing ruthenium surface sites (Figure 4d), but if all ruthenium sites were equally active, an increase is expected. Thus, these results clearly show that the depolymerization reaction is structure-sensitive. This hypothesis is further supported by the more pronounced changes in the crystallinity of total solids observed, where the usage of a catalyst with lower Ru loading and smaller metal particle sizes resulted in a lower melting point of the solids (Table S10, SI).

Next, the effect of H_2_ pressure was investigated. Formation of CP is catalyzed by the combined effects of H_2_ and the catalyst, so chemisorbed hydrogen species on the surface of the catalyst could play a role in facilitating the transformation of polymers to their monomers, involving C-heteroatom cleavage reactions.

Figure 5a illustrates the effect of H_2_ pressure on the hydro-depolymerization of nylon-6 over the 2.3 Ru/ZrO_2_ catalyst. Reaction conditions were identical to the experiments in Figure 4 at 350 °C and 2 h. The pressure here represents the hydrogen gauge pressure at room temperature. Depolymerization begins at atmospheric initial H_2_ pressure and results in approximately 37 wt % CP. It reaches its peak at the highest hydrogen pressure examined (36 bar H_2_ pressure at room temperature). Interestingly, a larger amount of HM is formed in response to increased H_2_ pressure. Apparently, a slower depolymerization of nylon-6 occurs at lower hydrogen pressure to produce CP. Partial pressure of hydrogen is a crucial factor that often affects hydro-depolymerization processes.

**Figure 5:**
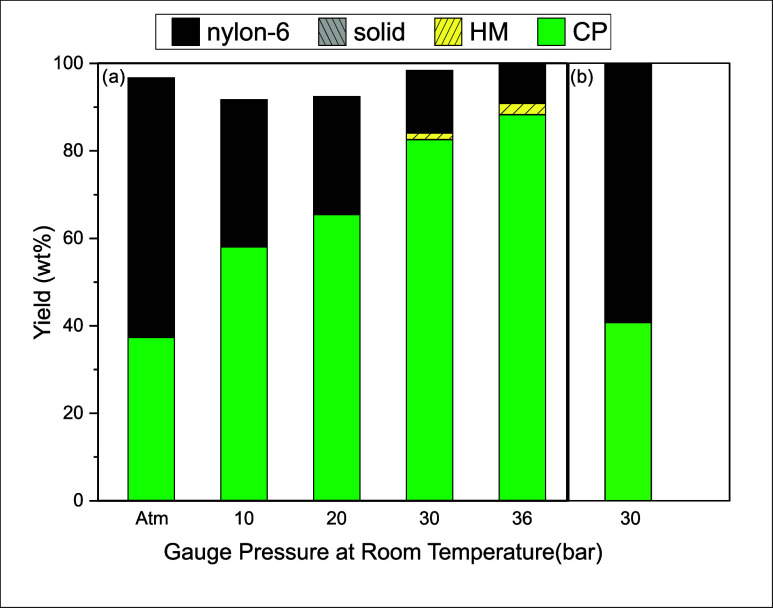
Nylon-6 depolymerization over the 2.3 Ru/ZrO_2_ catalyst (a) with varying H_2_ gauge pressures. (b) Alternative two-step approach comprising Step 1: catalyst pretreatment: solvent 57 g of hexadecane, 0.15 g of catalyst, temperature of 350 °C; pressure of 30 bar H_2_; treatment time of 2 h. Step 2: nylon-6 reaction with 30 bar gauge pressure of Ar. Reaction conditions: solvent = 57 g of hexadecane; feed = 3 g of nylon-6; polymer-to-catalyst mass ratio of 20:1; temperature of 350 °C; reaction time of 2 h. CP = ε-caprolactam; HM = hexamethylenimine.

The solubility of the hydrogen is related to its partial pressure, which in turn could influence the kinetics of the depolymerization reaction. The solubility of gas in liquids is governed by Henry’s law, and the literature shows that the Henry constant for hydrogen decreases with solvent molecular weight and follows the trend H_diols_ > H_alcohols_ > H_esters_ > H_aldehydes_ > H_ethers_> H_alkanes_.^54^ However, the long-chain alkane such as n-C_16_ has better hydrogen solubility (7.2 × 10^–4^ mole fraction at 1 atom and 25 °C) compared to polar solvents such as DMSO (0.7 × 10^–4^ mole fraction at 1 atom and 25 °C) or THF (2.7 × 10^–4^ mole fraction at 1 atm and 25 °C).^55^ Therefore, depolymerization of nylon-6 can still occur in this case at lower hydrogen pressures than those documented in the literature with DMSO and THF solvents using homogeneous catalysts.^24,25^ Apart from the influence of H_2_ solubilities on the depolymerization process, H_2_ can also play a crucial role in product selectivity. Several studies have demonstrated that elevated hydrogen pressure during the depolymerization of polymers such as polyolefins facilitates a greater degree of polymer fragmentation, thereby influencing selectivity outcomes.^10,35,56^ This occurs probably due to the adsorption/desorption equilibrium between H_2_ and other adsorbed species. In addition, hydrogen is also reported to have enhanced dehydrogenation–dehydration-type reactions.^57^ The results are in good agreement as the solubility of H_2_ is increased at high pressure, which is likely beneficial, as it makes hydrogen more available for adsorption on the metal sites. Unfortunately, as a negative response to increased pressure, a higher dehydrated product (i.e., HM) yield is seen. Apparently, the optimal conversion of nylon-6 to CP with low HM yield is achieved at a pressure of about 20 bar H_2_, where the selectivity is 100% to ε-caprolactam and the yield is 65%, or at 30 bar, where the yield is increased to 83%, with still a very high selectivity (98%).

Catalyst reduction followed by passivation, as used for catalyst pretreatment prior to the experiments in Figure 5a, likely forms a thin oxide layer, which H_2_ may further reduce, exposing the active metal during the reaction. This reduction of the oxide passivation layer may explain the role of H_2_ in nylon-6 depolymerization. To examine this hypothesis, we used an alternative two-step process for the removal of the oxide layer, and the results are shown in Figure 5b. In the first step, the reduced catalyst was treated in n-C16 at 350 °C for 2 h under H_2_ at 30 bar gauge pressure. This treatment was intended to ensure that the reduced and passivated catalyst underwent further reduction to remove the oxide layer, prior to its use for depolymerization. The use of n-C16 solvent prevented reoxidation of the catalyst when it was momentarily exposed to atmospheric air prior to the introduction of feedstock into the reactor. The second step involved conducting a depolymerization experiment wherein feedstock was added, and the reaction was performed under argon at 30 bar gauge pressure. This step was crucial for assessing the role of H_2_ in further reducing the oxide layer and its potential role in the catalytic reaction.

Analysis of the experimental results (Figure 5b) indicated that hydrogen’s role extends beyond the reduction of the catalyst and its passivating oxide layer. The CP yield was well below that with the catalyzed reactions and closer to that of the uncatalyzed reaction (blank), as shown in Figure 4. These results further indicate that H_2_ actively participates in the reactions, functioning as a catalyst instead of only promoting the metallic state of Ru during the reaction.

Considering the results from the previous experiments, the optimal condition of 350 °C with the 2.3 Ru/ZrO_2_ catalyst and 30 bar H_2_ pressure was selected for an investigation of the influence of reaction time on the depolymerization of nylon-6. The investigation was done with two different catalyst-to-polymer mass ratios to further enhance the understanding of the role of the catalyst in the depolymerization process. The experiment was conducted over varied reaction times from 1 to 6 h.

The yield of CP for a polymer-to-catalyst mass ratio of 20 is shown in Figure 6a (orange line). Data clearly show that the CP yield for a polymer-to-catalyst mass ratio of 20 starts from 31 wt % at a reaction time of 1 h and peaks at 94 wt % at 5 h before dropping significantly to 45 wt % after 6 h. However, HM yield, which was less than 1 wt % after the reaction at 1 h, reached a maximum of 5.4 wt % after 6 h of reaction with a concomitant decrease of the solids to less than 1 wt % (Figure 6b). This illustrates that formation of HM is a two-step reaction, where the intermediate CP eventually dehydrates to yield HM. Furthermore, the catalyst involvement becomes more evident when the quantity of the catalyst is increased.

**Figure 6:**
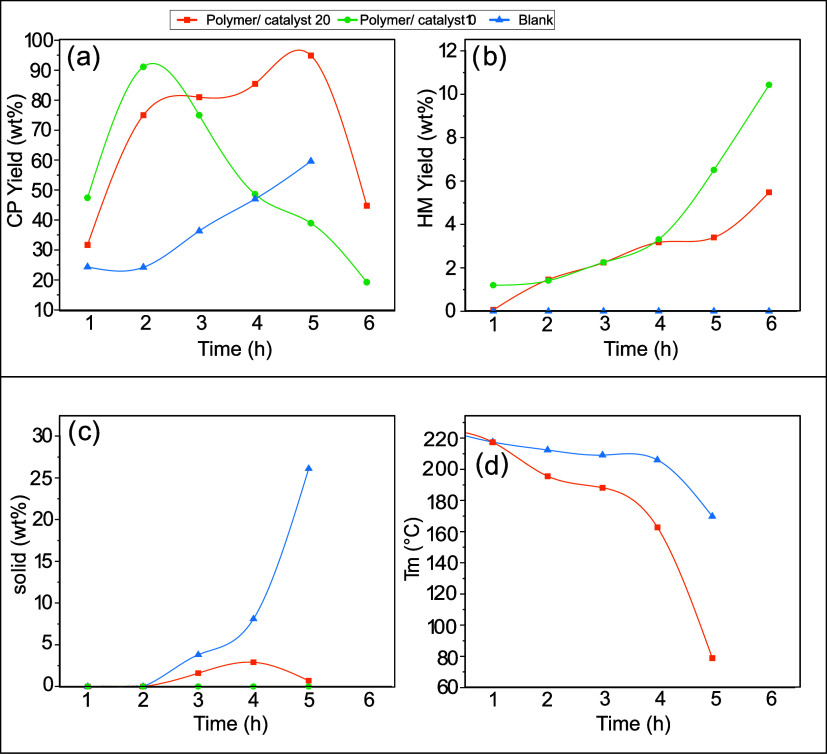
Yield of (a) CP = ε-caprolactam and (b) HM = hexamethylenimine, (c) solids, and (d) melting temperature (*T*_m_) of the total solids during nylon-6 depolymerization. Reaction conditions: solvent = 57 g of hexadecane; feed = 3 g nylon-6; polymer-to-catalyst mass ratio = ∞ (blue), 20:1 (orange), and 10:1 (green); temperature = 350 °C; pressure = 30 bar H_2_; reaction time = 1–6 h.

The trade-off between the catalyst-to-polymer mass ratio and the reaction time can be established from the results. The yield of CP for a polymer/catalyst ratio of 10 is shown in Figure 6a (green line). The yield of CP peaked earlier after 2 h with higher catalyst loading, whereas the yield of HM in this case increased significantly, reaching a maximum of 10.4 wt % at 6 h, again highlighting that HM formation involves a two-step reaction (Figure 6b). However, under the applied conditions and catalyst loading, a decrease in CP yield without a corresponding increase in HM yield, as shown in Figures 6a,b, during the first 4 h might suggest that CP undergoes decomposition into gaseous products over prolonged reaction times, indicating the occurrence of additional secondary reactions.

Some studies have examined the decomposition of CP with prolonged time or high temperature, especially during the hydrolysis of nylon-6.^58,59^ Therefore, it is important to optimize both temperature and time for the highest yield of CP. However, during the experiment, no significant change in the pressure was observed due to gas formation, but this might be due to the small amount of feed used. To understand this, an additional experiment was conducted with increased feed (9 g of nylon-6) and catalyst (0.45 g of 2.3 Ru/ZrO_2_), which again showed no significant changes in pressure after 6 h of reaction time. After the experiment, the reactor was cooled to ambient temperature, the headspace gas was released and passed through a scrubber filled with water. The flow of the gas was controlled by a needle valve, giving a rate of pressure release of about 2 bar/min. The analysis of the pH of the water from the scrubber showed an increase in pH, indicating the generation of water-soluble gaseous products, indicating that there indeed were some gaseous products, even though it was not a large amount that increased the pressure.

There are several reports in the literature regarding the decomposition of nylon and the formation of gaseous products. A literature review by Braun and Levin indicated that nylon-6 decomposes into gaseous products composed of CO_2_, H_2_O, hydrocarbons, and cyclopentanone when treated under vacuum at 400 °C. In the same review, it was also mentioned that hydrogen cyanide (HCN) and ammonia (NH_3_) were detected by heating polyamide (type not specified) at 350 °C in air as well as nitrogen.^60^ Furthermore, Rigby reported that the major gaseous product from nylon-6 decomposition is NH_3_ when heating at 250 °C for 40 min.^61^ Therefore, we suspect that CP and HM degradation follows similar pathways, producing mainly NH_3_, hydrocarbons, and CO gas with available H_2_. Gas was however produced only after prolonged reaction times or higher catalyst/polymer mass ratios; therefore, in this work, this will not be further discussed, since the main purpose was to investigate the hydro-depolymerization to CP. Moreover, the mass balance of the liquid and solid products during 2 h of the experiment was within 88–99% for all experiments with the catalyst, showing that the gas formation during these conditions was low.

Some degree of depolymerization of nylon-6 to CP can be observed even when H_2_ is used without any catalyst. The comparative blank reaction was conducted for a varying period of 1–5 h (Figure 6, blue line). It was found that with such conditions, the CP yield was 24 wt % at 1 h and increased to 60 wt % after 5 h of depolymerization. However, after 5 h, only 5.0 wt % of unconverted nylon-6 remains, whereas the yield of total solids reached 26.4 wt % (Figure 6c). Nylon-6, under uncatalyzed conditions, begins to convert into solids after 3 h, and a maximum of such solids was obtained after 5 h (Figures 6c, S10, and Table S11, SI).

The disruption of the polymer’s crystalline structure may be a prerequisite for C–N bond cleavage. A similar hypothesis has also been recently proposed by Sun et al. for C–C scission in polypropylene, where tacticity is a crucial prerequisite.^62^ Thus, the role of the catalyst and hydrogen can be further elucidated by examining the loss of the semicrystalline structure in the polymer during the course of depolymerization by analyzing the total solids collected after the reaction. In both catalytic and noncatalytic conditions, disruption in the crystallinity of the total solids begins at an early reaction stage (approximately at 1 h), as illustrated by melting point temperature (*T*_m_) vs time in Figure 6d. However, in the catalytic reaction (polymer-to-catalyst mass ratio of 20:1), the total solid’s crystallinity changes more quickly when the reaction time exceeds 1 h compared to the uncatalyzed reaction. After 3 h, in the uncatalyzed experiments, the total solids exhibit a decreased *T*_m_ (*T*_m_ = 209 °C), with a yield of 4 wt % of solids. Conversely, with the catalyst (polymer-to-catalyst mass ratio 20:1) and activated H_2_, *T*_m_ of the total solids decreases further to 188 °C within the same duration with only 2 wt % solids. This catalytic effect of decreasing *T*_m_ is comparable to that for the uncatalyzed reactions occurring beyond 4 h. Furthermore, the impact of the catalyst on the change in the crystallinity of the total solids is more pronounced with a decreased polymer-to-catalyst mass ratio, as evident by a further decrease in *T*_m_ after 1 h as compared to that for the higher polymer-to-catalyst mass ratio (Table S13, SI).

Further insight into crystallinity changes can be gained by examining the full width at half-maximum (fwhm) of the melting point peaks. After 1 h of reaction, the fwhm is 13 °C for noncatalytic conditions (Table S11, entry 1, SI) and 9 °C for catalytic conditions (Table S12, entry 1, SI), indicating quicker changes in the structure with the catalyst and hydrogen addition. Moreover, increasing H_2_ pressure from atmospheric to 36 bar leads to a further disruption of crystallinity (Table S14, SI). However, no significant change in crystallinity is observed when the catalyst is used with argon pressure instead of H_2_ for 2 h (compare Table S15, entry 01, with Table S14, entry 4, SI), suggesting that both the catalyst and hydrogen are necessary for altering the crystalline structure of the polymer.

On the other hand, the formation of solids (i.e., TFAA insoluble fraction) for both catalytic and noncatalytic experiments follows the same trajectory. The yield of solids was observed for both catalyzed and uncatalyzed reactions when the reaction time exceeded 2 h, likely due to prolonged high-temperature exposure, as illustrated in Figure 6c. Under catalytic conditions, significant conversion of nylon-6 to CP occurs before 3 h, resulting in minimal solid yield. Therefore, the use of the catalyst likely suppresses the formation of the main byproduct (i.e., solid) observed in the uncatalyzed reaction by effectively competing for the reactant. Efficient depolymerization apparently requires both crystallinity disruption and C–N bond cleavage, with the latter being the rate-limiting step. This is evident from the significant disruption of the semicrystalline structure, as illustrated by DSC analysis of solids collected after 5 h when only H_2_ is used without the catalyst, as shown in Figure 6d (also Table S11, SI). In this condition, CP yield remained lower than in the catalytic reaction, suggesting that both the catalyst and activated hydrogen play a role in C–N bond cleavage, thus also speeding depolymerization.

### 3.3 Influence of Plasticizers on Depolymerization

The robustness of catalytic depolymerization was evaluated using different plasticizers. The addition of plasticizers makes plastics more resistant to degradation and allows them to meet unique criteria for their applications. Thus, several plasticizers are used in nylon-6 to meet specific requirements. The most common plasticizer is water that reacts with ε-caprolactam via a ring-opening mechanism to form long-chain polymers. These long chains interact with one another through hydrogen bonding, forming sheet-like semicrystalline structures. Nylon-6 formed with this method is known to be hydroscopic in nature, which interacts with the crystalline form of nylon and affects its form via hydrogen bonding.^63^ This results in a decrease in strength; however, it also leads to an increase in toughness and flexibility and impacts chemical resistance.^64^ The water in nylon can affect the depolymerization in several different ways, including the hydrolysis of the amide linkage and the solubility of the polymers. Thus, we examine the depolymerization activity in the presence of water at different concentrations.

Figure 7 shows the yields for the depolymerization of nylon-6 after 2 h of reaction, at 30 bar H_2_ and 350 °C over 2.3 Ru/ZrO_2_. The water quantity in Figure 6 indicates the mass percentage of water relative to polymer mass; for instance, 10 wt % water is 3 g of nylon-6 and 0.3 g of water. The depolymerization product yields remain almost constant with an increase in the mass of the water. The reaction was conducted at a temperature below the critical point of water (*T*_c_ = 373 °C, *P*_c_ = 221 bar). At this near-critical temperature, the density of water transitions continuously from gas to the liquid phase. Moreover, the dielectric constant is usually between 2 and 30 at subcritical and supercritical temperatures, compared to 80 at ambient conditions. This difference in dielectric constant enhances the solubility of water with organic solvents.^65^

**Figure 7:**
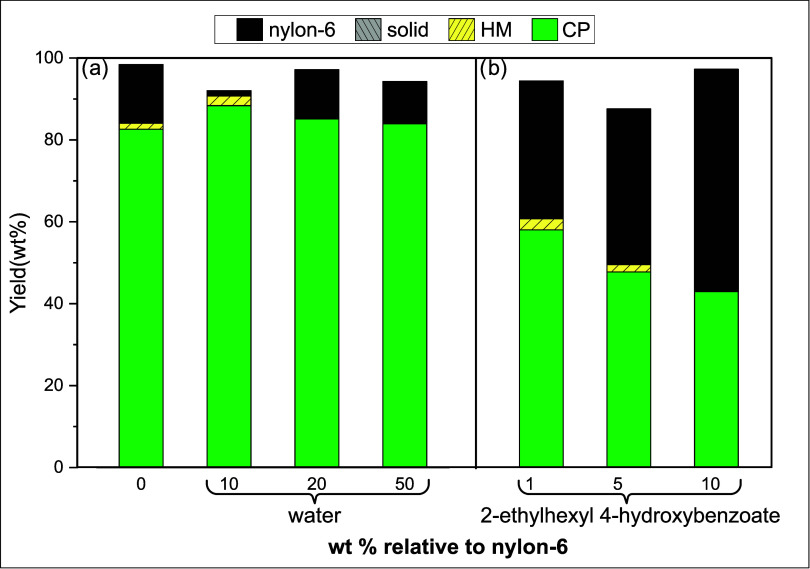
Nylon-6 depolymerization over the 2.3 Ru/ZrO_2_ catalyst with (a) water and (b) 2-ethylhexyl 4-hydroxybenzoate. Reaction conditions: solvent = 57 g of hexadecane; feed = 3 g of nylon-6; polymer-to-catalyst mass ratio = 20:1; temperature = 350 °C; reaction time: 2 h. CP = ε-caprolactam; HM = hexamethylenimine.

Water can also influence the depolymerization in several other ways. Nylon-6 is soluble in superheated water above 160 °C.^66^ Furthermore, water can form ions that aid in a variety of reactions that are catalyzed by acids and bases. The hydrogen bond between nylon-6 polymer sheets can be broken more successfully by ionic water.^67^ The ionic dissociation of water is endothermic; thus, it increases with an increase in temperature and is pKw ∼ 11 at 350 °C.^68^ It has been reported in various studies that the hydrothermal treatment of nylon-6 yields a mixture of CP and ε-aminocaproic acid at temperatures ranging from 300 to 400 °C and pressures of 200–350 bar.^58,69^ It is apparent that hydrothermal treatment needs harsh conditions or additional support by an acid catalyst.^70–72^ The increase in the CP yield in the presence of 10 wt % water and supercritical toluene has been reported by Kaiso et al. during an investigation with different organic solvents.^73^ The depolymerization to CP was inhibited by water content greater than 10 wt %, according to their investigation. Evidently, the process of cyclodehydration of ε-aminocaproic acid results in the formation of CP during hydrolysis. The presence of water may prevent CP formation by altering the equilibrium under the specified conditions. Recently, Coeck et al. reported that water content had a negative effect on the formation of dehydrated products during the ammonolysis of N-hexylhexanamide with the Nb_2_O_5_ catalyst, which was mainly due to the shift of the dehydration equilibrium to products such as hexanenitrile.^34^

Here, we found that the water content can slightly increase nylon-6 depolymerization up to 50 wt % water (compared to nylon addition), but it has no discernible effect on the yield of CP. Instead, high water content suppressed the yield of HM, which is likely caused by a negative effect of water on the dehydration of CP to yield HM. Furthermore, the greater impact of water can be seen in the crystallinity of nylon-6. For instance, with 10 wt % water in the feed, the *T*_m_ of the total solids decreased from 195 to 180 °C (Table S16, entry 1, SI), which explains a slight increase in the CP yield. Despite a slight elevation in *T*_m_ with 20 and 50 wt % water (Table S16, entries 2 and 3, SI) in the feed, there is no significant change in the CP yield.

Polyesters such as polyethylene terephthalate (PET) are another significant class of polymeric substances that are typically present in nylon as both an additive and an impurity. Often polyamide and polyesters are blended to create fabrics with high abrasion resistance. Figure 7b illustrates how 2-ethylhexyl 4-hydroxybenzoate (EHHB), which was chosen as a polyester model compound, affected the depolymerization process. Like for water, the wt % of EHHB added is relative to the nylon-6 feed. The conversion of nylon-6 decreased significantly, even with 1 wt % EHHB. However, the EHHB underwent complete destruction and resulted in products such as phenol, cyclohexane, alcohols, and other hydrocarbons. Several studies of the hydrogenation of benzoate have reported the formation of alcohols.^74,75^ During depolymerization, the aromatic ring is hydrogenated to yield cyclohexane. In addition, the successive hydrogenation and cleavage of ester bonds result in alcohols, phenols, and hydrocarbons. The yield of CP decreased from 83 wt % to 58 wt % with 1 wt % EHHB and further decreased to 43 wt % with 10 wt % EHHB added. Even at the low yield of CP at 1 wt % 2-ethylhexyl 4-hydroxybenzoate, the yield of HM was 1.8 wt %, which is higher than from the depolymerization without additives (1.5 wt % HM). An activity decline with a high concentration of EHHB (0.1 M per 0.1 mmol of the amide bond) during ammonolytic hydrogenation of N-hexylhexanamide after 5 h reaction time was reported by Coeck et al.^34^ The activity decline is due to the competitive C–N cleavage of the polyamide and C–O cleavage in polyester over the catalyst. Adsorption of esters on the support can result in a weaker bond between polyamide and the catalyst, thus suppressing the depolymerization process. Additionally, the hydrogenation of ester reduces the efficiency of adsorbed hydrogen species in the formation of CP. This competitiveness can be noticed easily in the analysis of total solids collected after the reaction, which exhibited a melting point much higher compared to the EHHB-free reaction under the same conditions (Table S17, SI).

### 3.4 Catalyst Reusability Test

The regeneration of the activity of the catalyst was evaluated. Based on the TGA results of total solids along with the catalyst (Figure S12a,b, SI), a relatively high temperature (400 °C) is required to remove the solid residue. Thus, the catalyst was calcined at 400 °C in air and subsequently reduced in H_2_ to regenerate its activity. To compensate for losses during calcination and filtration, 20 wt % (0.03 g) of fresh catalyst was added for each experimental run before reduction in H_2_, such that the total amount of catalyst was the same (0.15 g) for all the experiments. We estimate that the added catalyst may have reduced the observed decrease in CP yield by at most 2 percentage points, based on applying the same relative loss in activity observed for the reused catalyst to the fraction of the fresh catalyst added. CP yield dropped by 14% after the first reuse cycle and remained almost the same for the second reuse cycle, whereas HM yield increased slightly by 1 wt %. (Figure 8).

**Figure 8:**
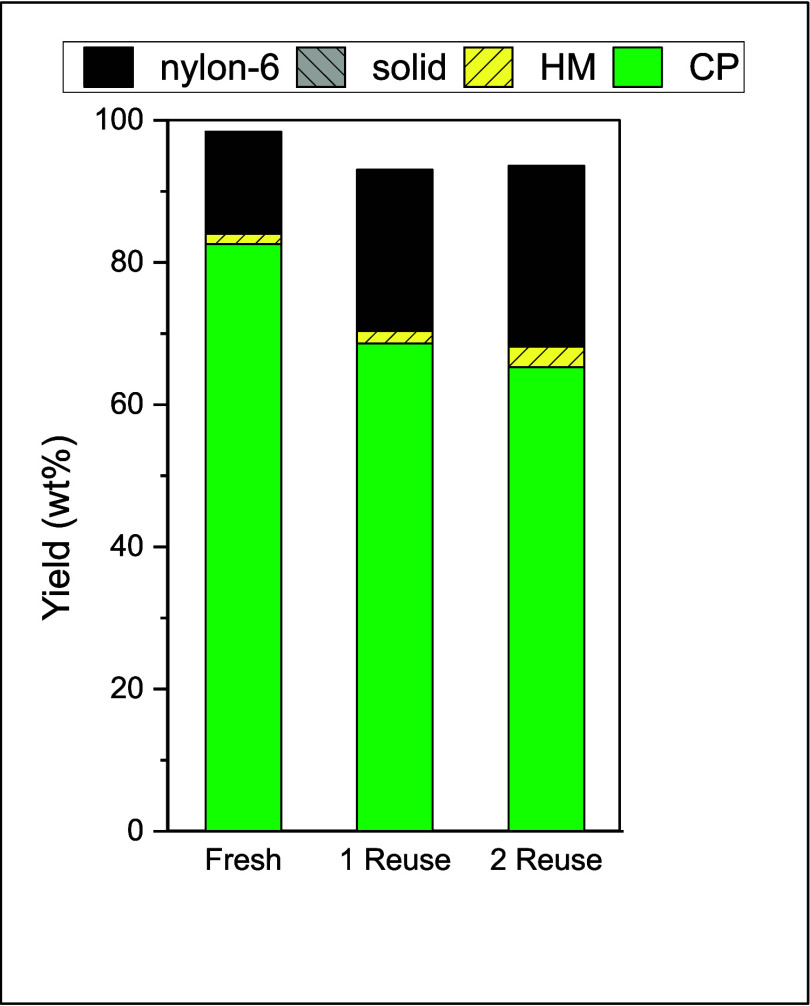
Reusability test of the catalyst. Reaction conditions: solvent = 57 g of hexadecane; feed = 3 g of nylon-6; polymer-to-catalyst mass ratio = 20:1. Regeneration conditions: 400 °C for 5 h, 0.12 g of the spent catalyst and 0.03 g of the fresh catalyst for reuse 1 and reuse 2.

TGA analysis was performed to assess potential solid deposits on the regenerated catalyst. The TGA results for the catalyst before the first and second reuse cycles (Figure S12c, SI) showed no significant differences, indicating that calcination effectively removed most solid deposits. However, visual inspection revealed a few residual nylon-6 particles, suggesting that some may be resistant to complete removal by calcination. Further characterization using nitrogen physisorption showed a decrease in the surface area of the regenerated catalyst from 59 to 50 m^2^/g after the first use cycle and to 48 m^2^/g after the second reuse cycle. This decline suggests possible pore blocking or residual solid deposits, which could contribute to the observed decrease in catalytic activity.

### 3.5 Proposed C–N Cleavage Mechanism

The process by which nylon-6 hydro-depolymerizes is comparable to amide bond hydrogenation. The electronic stability of carboxamide, which is related to the difference in electronegativity between nitrogen and oxygen lying next to each other in the amide bond, is typically the cause of its resistance to chemical reactions. This arrangement ensures the existence of resonance properties due to the charge distribution between the nitrogen and oxygen atoms. The short C–N bond compared to amines and the long C–O bond compared to other carbonyl compounds are caused by donor groups such as alkyl groups present in amides next to the carbonyl group.^76^ Due to the presence of 6 lone pairs of electrons, the O–C–N moiety exhibits 3 possible resonance structures that cause electron delocalization. A stronger C–N bond and a reduction in the electrophilicity of carbon are favored by the electron-donating groups next to the nitrogen atom, resulting in hindrance to nucleophilic additions. Alkyl groups typically have a greater electron-donating effect than hydrogen due to the inductive effect. This is another explanation for the lower reactivity of polyamides to nucleophilic attacks compared to primary amides. Furthermore, polar compounds likely influence electron density via the formation of hydrogen bonds. Nonpolar solvents inhibit this interaction and thus minimize the interaction effects. However, under appropriate conditions, most amides undergo hydrogenation, which proceeds via the addition of hydrogen to the amide to afford the hemiaminal intermediates.^77^ These intermediates are short-lived and undergo C–O cleavage to eliminate amines.^78^ Alternatively, hemiaminal intermediates can also undergo C–N cleavage to give lower amines and alcohols.^79^

The C–N cleavage of polyamide in the presence of H_2_ and a catalyst could proceed similarly to the C–N of amides via the formation of hemiaminal intermediates. Depolymerization requires harsh conditions due to the restrictive behavior of the amide bond, as already explained. The early studies of the depolymerization of nylon demonstrated the need for either a strong acid or a strong base to break the polymer chain.^80^ Recently, it was shown that hydro-depolymerization proceeds via C–N cleavage, yielding diamines, diols, and amino alcohols.^24^ It has been shown that depolymerization generally occurs in the presence of molecular hydrogen and an active metal catalyst.^24^ Scheme 3 shows the proposed mechanism of the reaction as observed in this study. The depolymerization in the absence of the catalyst takes place either via the backbiting mechanism or the INCH worm movement. The INCH worm movement is much slower at low temperatures, where backbiting occurs by about 2 orders of magnitude greater rate at 350 °C.^81^ So, the dominant mechanism at 350 °C should be backbiting, where internal hydrogen migration from the end group results in simultaneous cleavage and cyclization to form CP, as shown in Scheme 3a.^81^ Similarly, this kind of mechanism was demonstrated via DFT studies by Wursthron et al. using 6-amino-*N*-methylhexanamide as a model compound. In their findings, they showed how a tris­[bis­(trimethylsilyl)­amido] lanthanide catalyst works as an anchor for this backbiting mechanism, resulting in faster conversion of the nylon. However, in our study, the depolymerization using the backbiting mechanism, in the absence of Ru/ZrO_2_ or activated hydrogen, is slower than the catalyzed reaction at 350 °C, causing the polymer chain to reorganize and form other solid byproducts. When a catalyst is used (in this case, Ru with adsorbed hydrogen), internal hydrogen migration is no longer necessary, which likely accelerates the depolymerization process. Once the hemiaminal structure is formed with the addition of hydrogen, the electronic structure becomes unstable primarily because fewer lone pairs of electrons from nitrogen contribute to the carbonyl group. The cleavage of the C–N bond is triggered by this instability. With a lone pair of electrons, the nitrogen atom is a more potent nucleophile and targets the carbon at the opposite end of the monomer, as depicted in Scheme 3b, to eventually cyclize and produce CP. Hydrogenative C–N cleavage followed by cyclization is likely driven by ring stabilization energy. This is the common cyclization mechanism observed during the dehydration and cyclization of 6-aminocarpoic acid to CP.^82,83^ In addition, the formation of 6-aminocapronic acid as an intermediate was observed by several studies during the hydrolysis of nylons.^58,59^ However, in our case, 6-aminocapronic acid was not detected, even at short reaction times, indicating that the concurrent cleavage of the C–N bond and cyclization is the dominating mechanism, as suggested in Scheme 3.

**Scheme 3:**
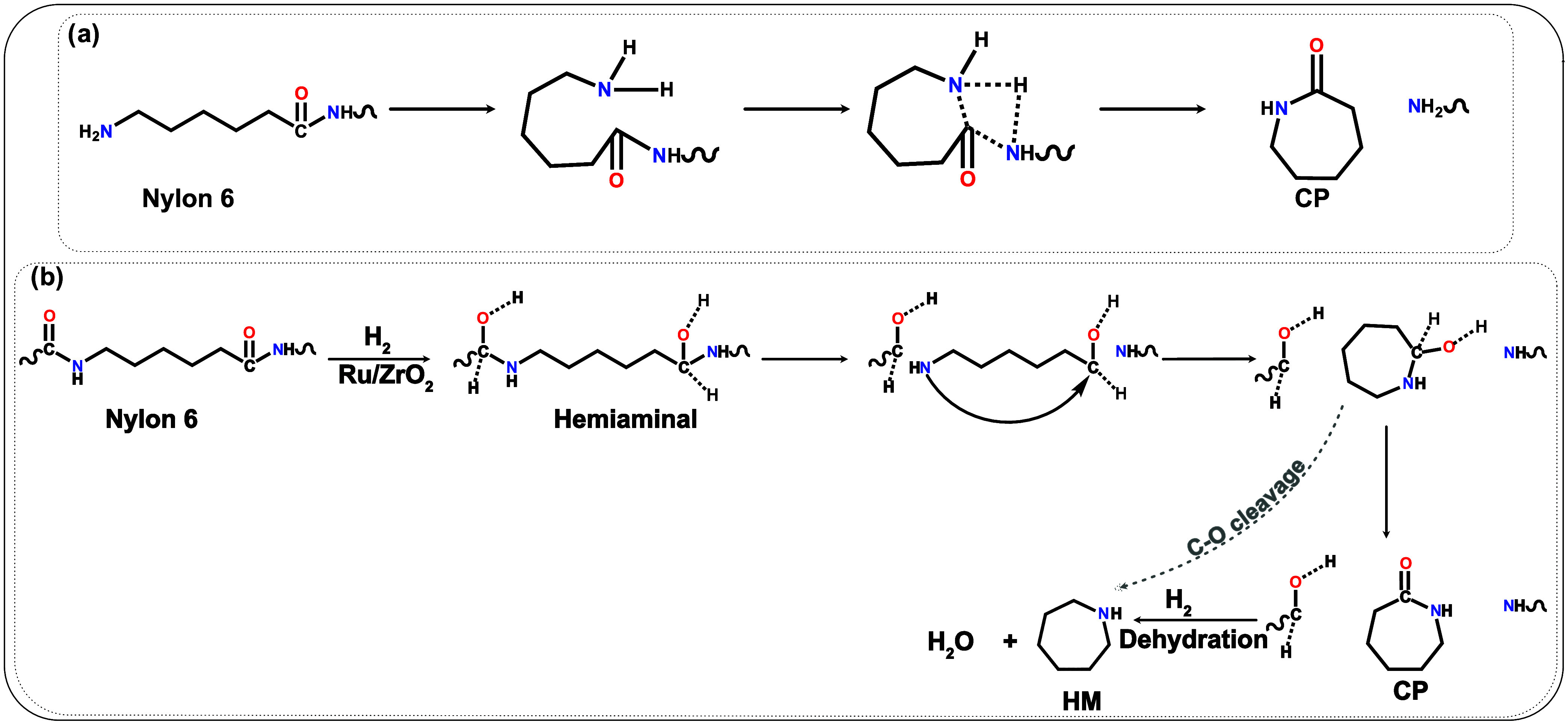
Proposed Reaction Mechanism^a^

a (a) Thermal depolymerization of nylon-6 via the backbiting mechanism.^81^ (b) Catalytic hydro-depolymerization of nylon-6 over Ru/ZrO_2_ to ε-caprolactam­(CP) and hexamethyleneimine (HM).

## 4 Conclusions

In this study, ruthenium nanoparticles on zirconia (2.3–6.9 wt % Ru/ZrO_2_) with activated hydrogen were shown to be an effective heterogeneous catalyst for hydro-depolymerization of nylon-6. At 350 °C, 30 bar H_2_, and with hexadecane as a solvent, excellent depolymerization activity was demonstrated to produce the industrial monomer ε-caprolactam. ε-Caprolactam yields of over 94 wt % were achieved for depolymerization of nylon-6 (350 °C, 30 bar H_2_ and 5 h). Under a shorter reaction time (350 °C, 30 bar H_2_ and 2 h), the ε-caprolactam yield was 82 wt %. Through rigorous quantification of the products, this study identified a trade-off between the polymer–catalyst mass ratio and reaction time to optimize ε-caprolactam yield at the expense of other byproducts. This study also related catalytic activity with metal loading, where a low metal loading of 2.3 wt % Ru, bearing a particle size of 9.5 ± 2.34 nm, demonstrated the most efficient catalyst, indicating that depolymerization of nylon-6 is sensitive to the structure of the catalyst. XPS and H_2_-TPR revealed the greater presence of reducible species of Ru with decreased metal particle size. It was observed that depolymerization progresses with a change in crystallinity prior to C–N bond cleavage, where catalyst and hydrogen both play a dramatic role to provide dual functionality. We propose that this dual functionality of the catalyst and H_2_ causes simultaneous changes in the structure of the polymer, followed by C–N bond cleavage, which affords cyclization, producing CP. The catalytic system is also compatible with nylon-6, containing up to 50 wt % water. However, the activity of the catalyst was suppressed toward nylon-6 depolymerization by the presence of esters such as benzoates. The experimental mechanistic reasoning suggests that the C–N cleavage under the applied conditions occurs via hemiaminal formation, which facilitates hydrogen migration. These findings can potentially be applied to real nylon-6 waste containing contaminants, paints, and other types of fillers, but additional research is needed to explore these possibilities.

## Supplementary Material



## Notes

Additional experimental data, product analysis, and catalyst characterization (PDF)
