# Supplementary material for: Depolymerization of Nylon‑6 over a Supported Ruthenium Catalyst to ε‑Caprolactam
Source: ACS Environ Au. 2026 Apr 9;6(4):589–607. doi: 10.1021/acsenvironau.5c00272 (PMC13377514; doi:10.1021/acsenvironau.5c00272)
Supplement: Supplementary file 1 [file vg5c00272_si_001.pdf]

Supplementary information

Depolymerization of nylon-6 over supported ruthenium catalyst to ε-caprolactam

Prabin Dhakal, Abdenour Achour, Phuoc Hoang Ho, Aqsa Noreen, Derek Creaser, and Louise Olsson

Chemical Engineering, Competence Centre for Catalysis, Chalmers University of Technology, 412 96 Gothenburg, Sweden

1. Catalyst characterization

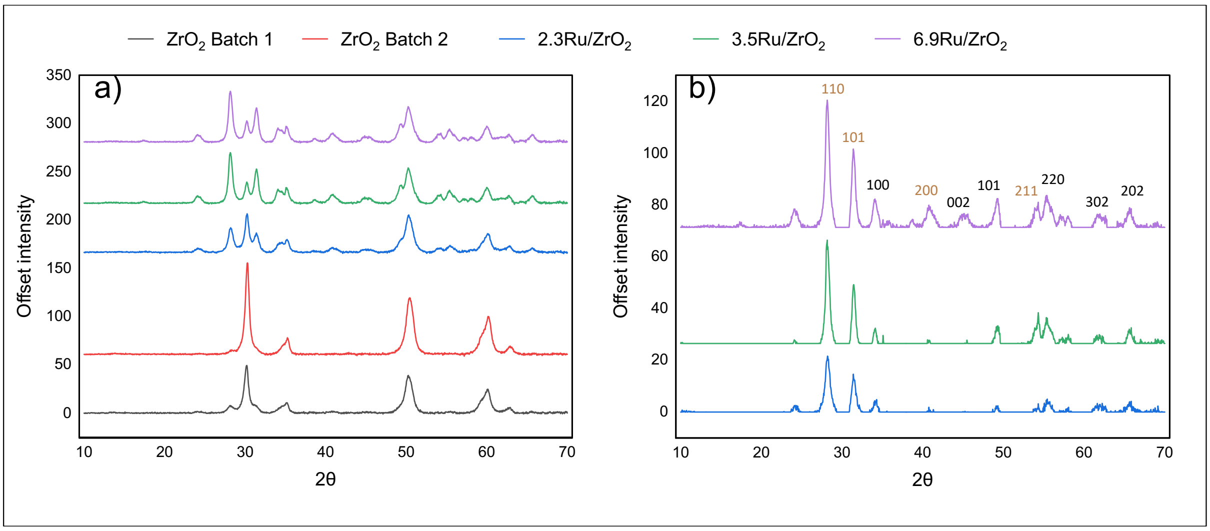

Figure S1. XRD diffractogram of catalyst samples a) as obtained and b) after subtraction of ZrO_2 from Ru loaded samples.

Table S 1. Summary of H_2-TPR: Degree of reducibility based on assumption that all Ru initially present as RuO_2.

**Catalyst**	**H_2 consumption (µmol/g of catalyst)**	**Degree of reducibility (%)**
2.3Ru/ZrO_2	86.4	19.0
3.5Ru/ZrO_2	129.0	18.6
6.9Ru/ZrO_2	199.1	14.5

Table S 2. Relative area of peaks from CO-DRIFTS results as per the peak assignment in Figure S3.

	**Relative Peak area (%)**				
**Catalyst**	Ru_2-CO	Ru-CO	Ru-$(CO)_2$	$Ru_{1.3}$-$(CO)_3$	**Stoichiometric factor**
2.3Ru/ZrO_2	37.9	36.2	21.6	4.3	1.3
3.5Ru/ZrO_2	41.5	27.7	26.2	4.6	1.3
6.9Ru/ZrO_2	40.9	13.6	36.4	9.1	1.3

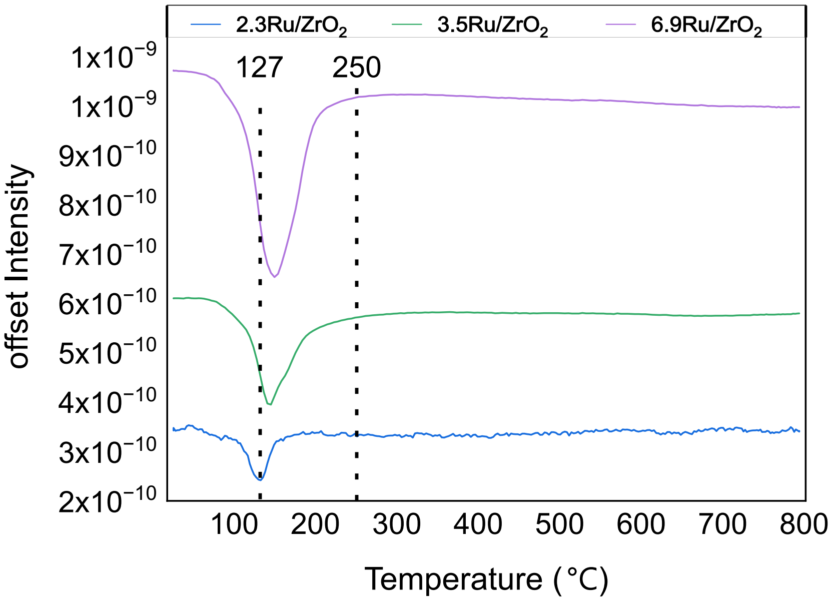

Figure S2. H_2-TPR of the catalysts (25-800 °C).

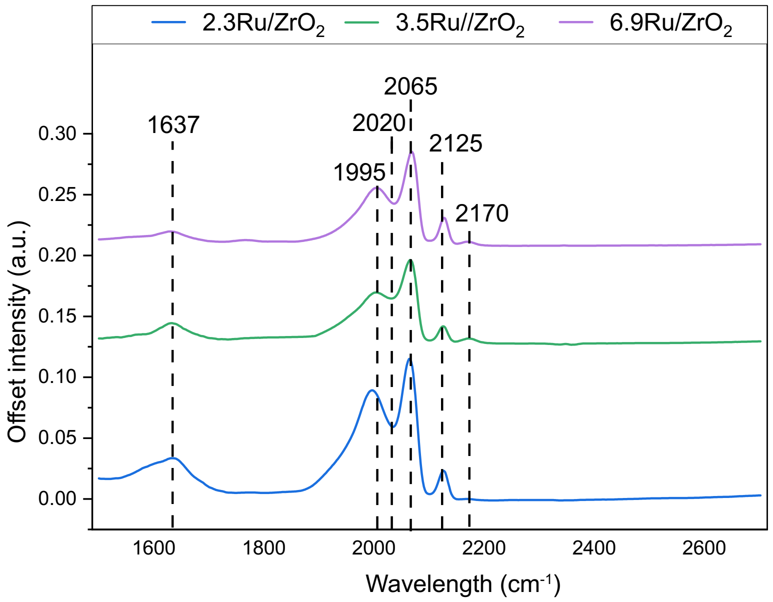

Figure S3. CO-DRIFTS at 35 °C of the catalysts.

Table S 3:Summary of results of CO-chemisorption for Ru catalysts

**Catalyst**	**Dispersion (%)**	**Surface Ru (mol)***	**Crystallite Size**	
			hemispherical (nm)	cube (nm)
2.3Ru/ZrO_2	14.1	0.003	9.5	7.9
3.5Ru/ZrO2	11.2	0.004	11.9	9.9
6.9Ru/ZrO2	7.4	0.005	21.1	18.1

$* \, moles\ of\ surface\ Ru = \ Dispersion \times Loading\ (in\ moles)$

Table S4. Deconvoluted area of XPS spectrum.

**Catalyst**	**$3d_{5/2}$**		**$3d_{3/2}$**		**Total**
	**Ru^0**	**RuO_2**	**Ru^0**	**RuO_2**	**Ru^0 (%)**
2.3Ru/ZrO_2	340.4	305.1	228.1	204.4	52.3
3.5Ru/ZrO_2	259.8	94.4	174.1	63.23	73.4
6.9Ru/ZrO_2	991.7	415.8	664.4	278.6	70.4

2. Nylon-6 characterization

Table S5. Result of Elemental analysis and DSC analysis (shown in figure S8a) of nylon-6.

**Elemental Analysis**			**DSC analysis**	
**Nitrogen [wt%]**	**Carbon [wt%]**	**Hydrogen[wt%]**	Tm(°C)	FWHM(°C)
12.2	63.8	10.8	226.3	11.8

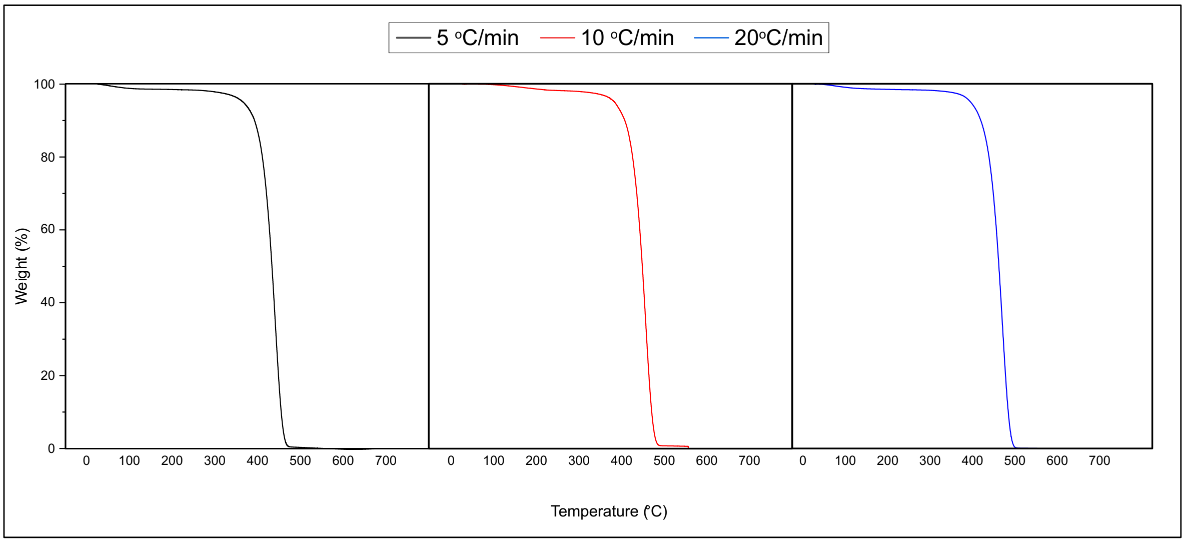

Figure S4. TGA of nylon-6 at different heating rates under a constant flow of N_2 at 50 Nml/min.

3. CP and HM identification

a) identification by NMR spectroscopy

The products soluble in acetone were examined by nuclear magnetic resonance (NMR) using 1,4-dinitrochlorobenzene as an internal standard. Aliquots of dry samples obtained after the removal of acetone were dissolved in chloroform-d6, with 1H NMR showing a signal of the proton bound to the methylene group of the rings of caprolactam between 1.3 and 4 ppm, as shown in the Figure S5. The appearance of the proton bound to the amide group (NH) varied depending on the dilution level of the sample. The spectrum was further compared with the predicted 1H spectrum from ChemDraw. Additionally, the sample structure was confirmed using 2D HSQC NMR spectroscopy as shown in the Figure S6. The HSQC spectrum showed the corresponding carbon associated with protons from the proton NMR. The amide proton was not associated with any carbon whereas there was a carbonyl carbon above 150 ppm (not shown in the Figure S6) confirming the structure of caprolactam in the sample.

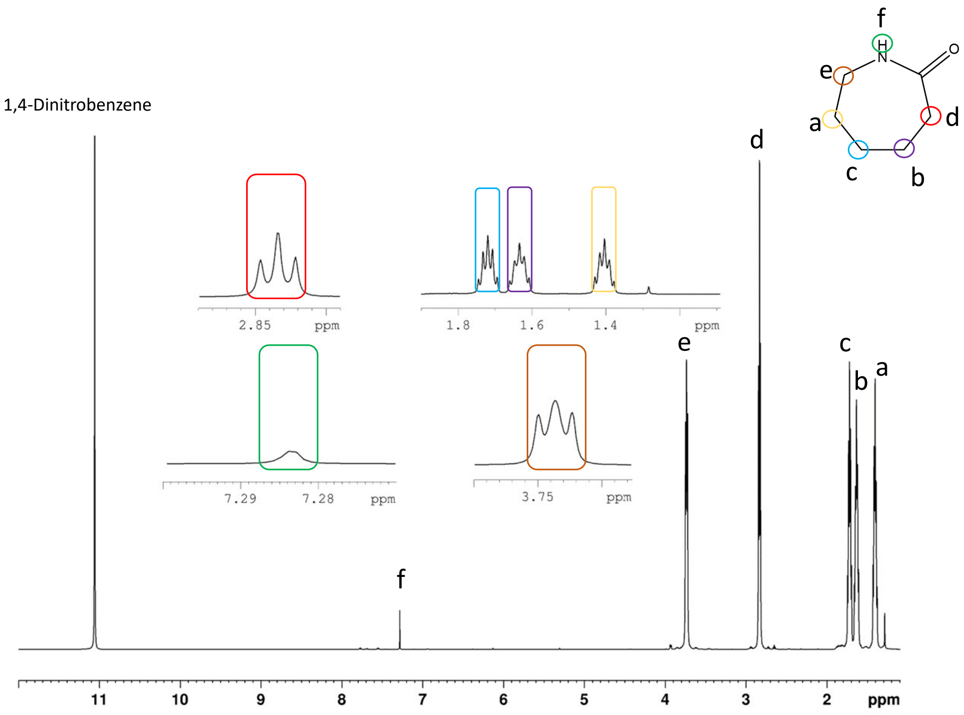

Figure S5. Proton NMR of acetone phase sample with 1,4-dinitrobenzene as internal standard.

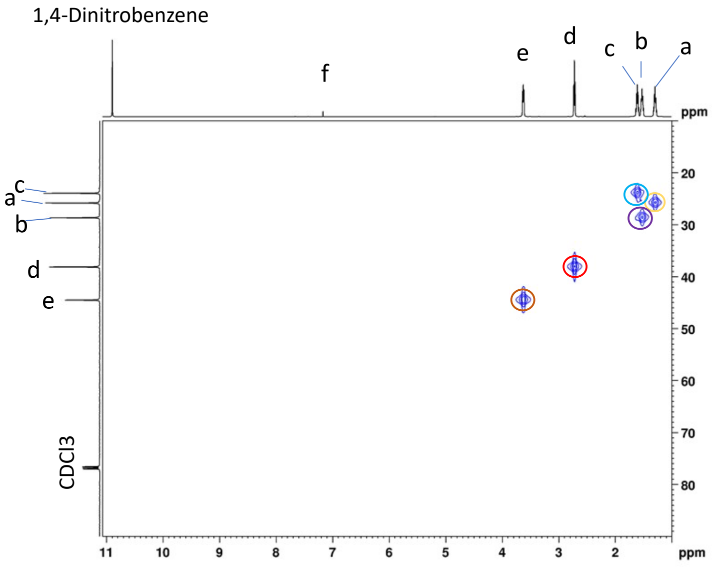

Figure S6. HSQC NMR of acetone phase sample showing corresponding carbons and proton.

b) MS fragmentation pattern

CP (Caprolactam) (M/w= 113 g/mol)

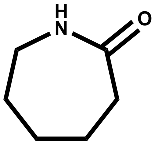

GC-MS (EI, 70ev): m/z (relative Abundance, %) 113 (100), 85 (58), 84 (50), 67(12), 56 (68) 55 (78), 43 (11), 42 (35), 41(36), 39 (24).

HM (hexamethyleneimine) (M/w=99 g/mol)

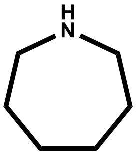

GC-MS (EI, 70ev): m/z (relative Abundance, %) 99 (61), 98 (28), 84 (16), 71(8), 70(100), 56 (47), 44 (21) , 43(58), 41 (22), 39 (15).

c) DSC Melting temperature peak of CP

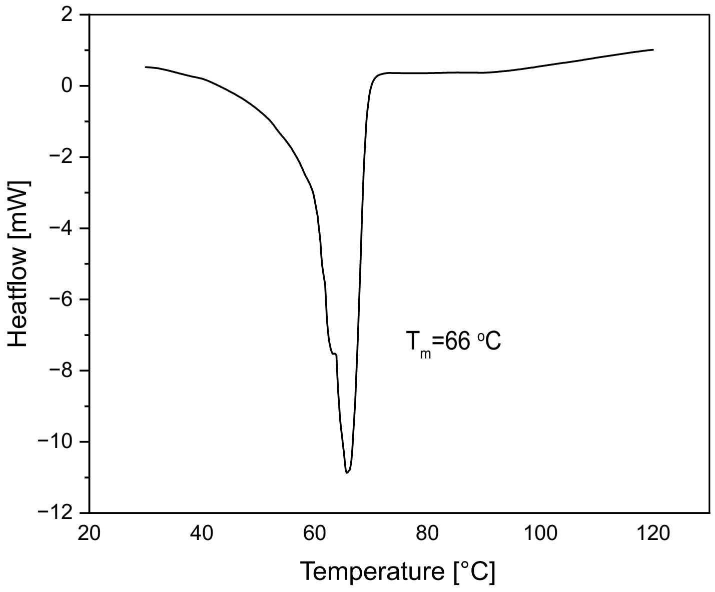

Figure S7. DSC analysis of the caprolactam obtained as product after 5 h of reaction. Analysis conditions: heating rate of 10°C/min with constant purge of N_2 at 50 Nml/min.

Table S6. GC-area comparison of hydrocarbons components from reactions conducted without and with nylon-6. Reaction conditions: catalyst: 0.15g, hexadecane solvent: 57 g, 30 bar hydrogen, reaction time: 5 h

RT [min]	Name	Only C16 Area %	C16+Nylon-6 Area %
2.496	C4	0.75	0.73
2.642	C5	3.23	2.91
3.008	C6	6.35	5.94
3.873	C7	7.68	8.91
5.788	C8	9.23	10.40
7.702	C9	9.01	10.60
9.165	C10	11.41	12.07
10.299	C11	10.39	12.59
11.22	C12	10.65	12.58
12.018	C13	11.78	13.52
12.762	C14	19.50	9.75

Table S7. Monomer distribution in acetone and hexadecane. Reaction conditions: nylon-6 (mw-10,000)-3g, catalyst: 0.15g, hexadecane solvent: 57 g, 30 bar hydrogen, reaction time: 5 h. Basis of calculation: total quantified monomers.

	**Temperature (°C)**	**% of monomers yield in acetone phase**	**% of monomers yield in hexadecane phase**
2.3Ru/ZrO_2	350	94.9	5.1
Blank	350	94.1	5.9
2.3Ru/ZrO_2	300	85.7	14.3
Blank	300	33.3	66.7

4. **Residual solid analysis**

Nylon-6 is typically soluble in TFAA in its semicrystalline form. The changes in its crystalline form were tracked using DSC analysis of the total solids. The total solids with a higher degree of crystallinity exhibit relatively higher melting point temperatures (T_m) in the DSC analysis, because a high amount of energy is required to disrupt the well-organized crystalline structure. Conversely, solids with lower crystallinity display a shift towards lower T_m, reflecting the reduced energy needed to disrupt their less ordered structure.

Upon analysis, it was found that nylon-6, which had significantly changed its semicrystalline form, remained undissolved in TFAA. After experiments at 250°C and 300°C, the total solids were fully soluble in TFAA, confirming their semicrystalline nature. However, their analysis showed a lower T_m in contrast to pure nylon-6, indicating some changes in the structure of the nylon-6. (**Figure S8, SI**). Analysis of the total solids showed no significant changes in their elemental composition (**Table S7, SI**) compared to that of nylon-6. However, solids from the uncatalyzed reaction at 350°C, and those with argon instead of hydrogen, were insoluble in TFAA, with T_m below 190°C. Similar behavior from nylon-6.6 in the literature, resulted in dark insoluble materials, and was explained to be due to the cross-linking of the polymers during thermal degradation.(1).. The cross-linking of functional groups such as NH with C=O led an increase in activation energy that slowed the depolymerization activity.(2).. Insolubility of the total solids indicates inaccessibility of their amide linkage for TFAA, thus this was used as a benchmark for conversion of nylon-6. In other words, the fraction of total solids soluble in TFAA was considered as unconverted nylon-6 (Figure 3) and the remaining fraction was considered solids as shown in Figure 3.

All TGA analyses were performed under a constant nitrogen purge of 50 NmL/min. Approximately 2 mg of the sample was heated from room temperature to 800 °C at a ramp rate of 10 °C/min and measurements were performed using a TGA device from Mettler Toledo. DSC was performed using DSC 2, with samples heated from room temperature to 300 °C at a rate of 10 °C/min in a nitrogen flow of 50 NmL/min. The pure nylon-6 showed a melting point at 226 °C indicated by the minimum in Figure S8a

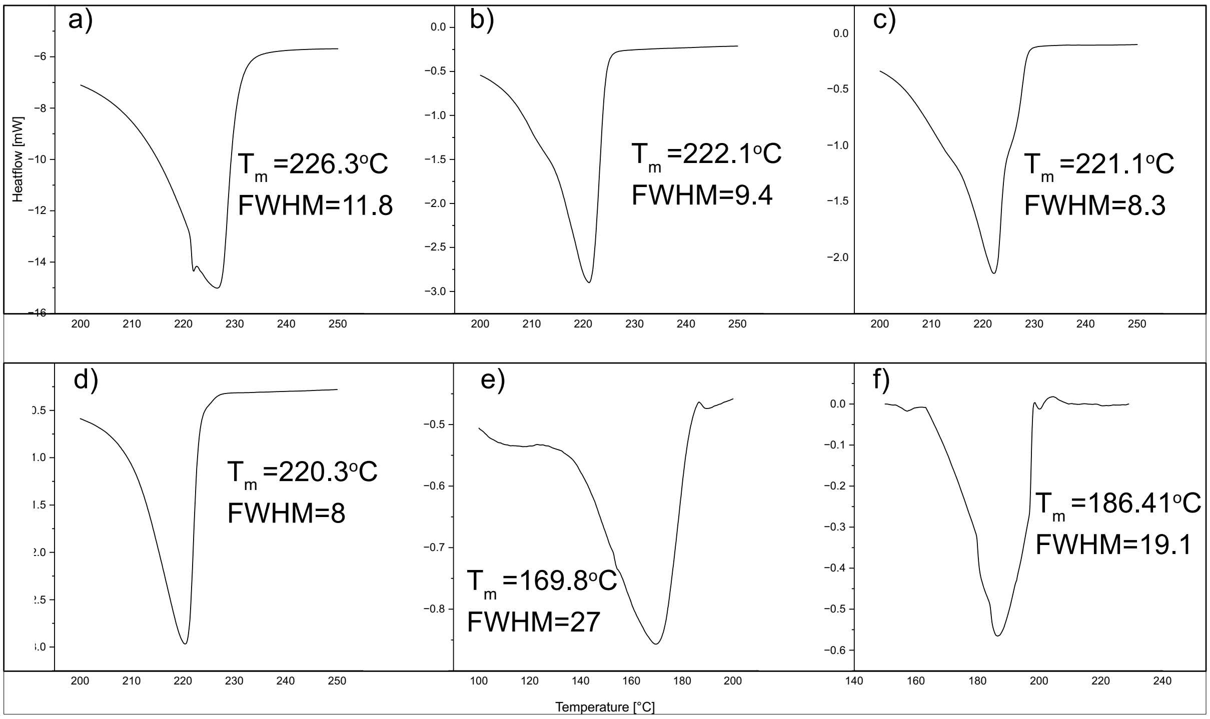

Figure S8. DSC analysis of the solids at heating rate of 10°C/min with constant purge of N_2 at 50 Nml/min a) Nylon-6, b) solids after experiment at 250 °C with catalyst, c) Solids after experiment at 300 °C with catalyst, d) Solids after experiment at 300 °C without catalyst, e) Solids after experiment at 350 °C without catalyst and f) solids after the experiment with catalyst at 350 °C and 30 bar Argon.

Table S8. Elemental analysis of solids after reaction. Reaction conditions: nylon-6 (mw-10,000)-3g, catalyst: 0.15 g, hexadecane solvent: 57 g, 30 bar hydrogen, reaction time: 5 h.

	**Temperature (°C)**	**Nitrogen [wt%]**	**Carbon [wt%]**	**Hydrogen [wt%]**
2.3Ru/ZrO_2 [(a)]	350	10.3	60.3	10.2
Blank [(b)]	350	10.7	65.5	10.9
2.3Ru/ZrO_2 [(b)]	300	11.9	62.7	10.9
Blank [(b)]	300	12.0	63.7	11.1
2.3Ru/ZrO_2[(b)]	250	11.7	60.9	10.4
Blank[(b)]	250	11.2	60.2	10.8

[(a)]– 30 bar Argon, [(b)] – 30 bar hydrogen.

Result of the repetition of experiments with 2.3Ru/ZrO_2 based on the experimental protocol in Figure 4.

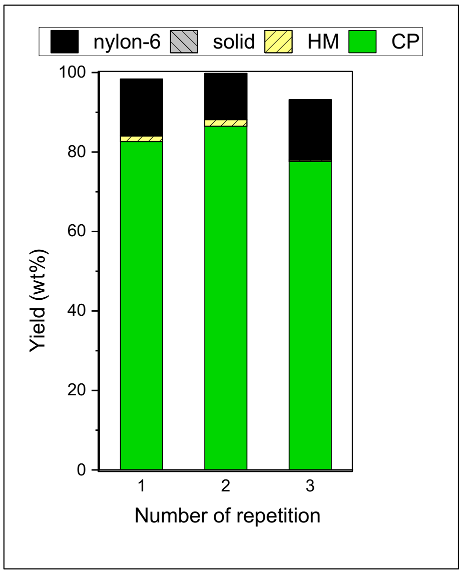

Figure S9. Reproducibility test for nylon-6 depolymerization over 2.3RuZrO2. Reaction Conditions: solvent = 57 g of hexadecane; feed = 3 g nylon-6; polymer to catalyst mass ratio = 20:1; temperature=350 °C; pressure = 30 bar H_2; reaction time: 2 h. CP = ε-caprolactam , HM = hexamethyleneimine.

Table S9. Product yields as per the experiments conducted in Figure S9.

**Repetitions**	**CP (Wt%)**	**HM (wt%)**	**nylon-6 (wt%)**
**1**	**82.6**	**1.5**	**14.3**
**2**	**86.5**	**1.6**	**11.7**
**3**	**77.6**	**0.4**	**15.2**
**standard deviation**	**4.46**	**0.67**	**1.85**

Table S10. DSC Analysis of residual solid obtained as per experiment protocol in Figure 5.

**Catalyst**	**T_m (°C)**	**FWHM(°C)**
Blank	212.4	9.9
2.3Ru/ZrO_2	195.7	22.1
3.5Ru/ZrO_2	204.1	14.2
6.9Ru/ZrO_2	205.8	13.6

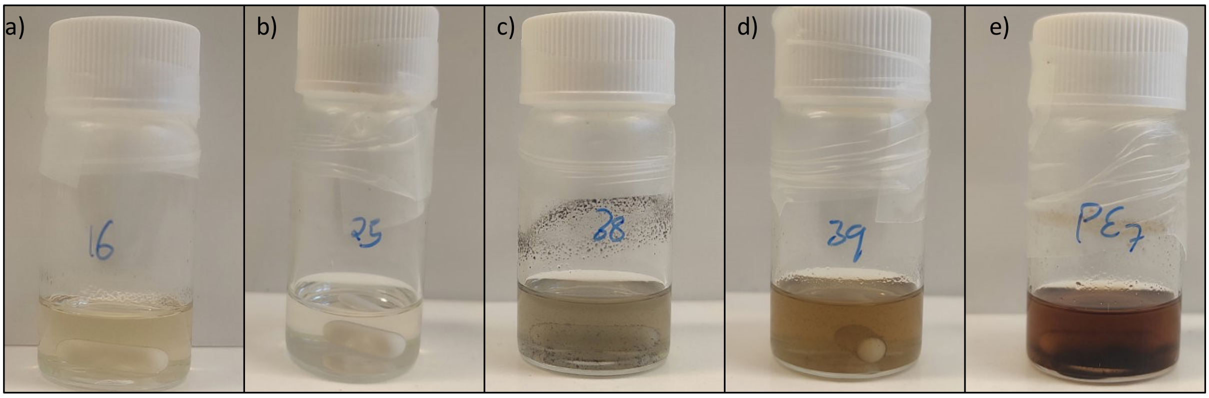

Figure S10. solids after treatment with TFAA collected after the uncatalyzed reaction at 350 C, 30 bar H_2 Reaction time of a) 1 h b) 2 h c) 3 h d) 4 h and e) 5 h.

Table S11. Fractions of TFFA soluble and insoluble total solids after uncatalyzed reaction and corresponding melting point temperature and FWHM determined by DSC for reaction time of 1-5 h.

Time (h)	Total solids yield (wt%)	Soluble fraction (wt%)[a]	insoluble fraction (wt%)[a]	T_m (°C)[b]	FWHM(°C)[b]
1	67.3	100.0	-	217.5	13.4
2	62	100.0	-	212.4	9.9
3	53.7	92.8	7.2	209.1	9.1
4	42	80.7	19.3	206.0	11.2
5	31.4	15.9	84.1	169.8	27

[a]-determined using TFAA functionalization method.

[b]-determined using DSC results

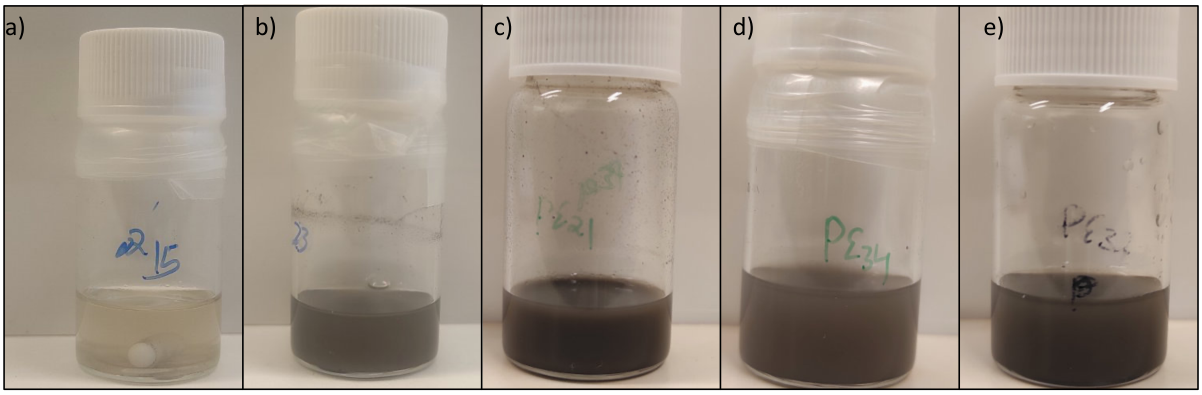

Figure S 11: solids after treatment with TFAA collected after the catalyzed reaction at 350 °C, 30 bar H2, Reaction times of a) 1 h, b) 2 h, c) 3 h, d) 4h, and e) 5h.

Table S12. Fractions of TFFA soluble and insoluble total solids after catalyzed reaction (2.3Ru/ZrO_2), and corresponding melting point temperature, and FWHM determined by DSC for reaction times of 1-5 h for polymer-to mass ratio 20:1.

Time (h)	Total solids yield (wt%)	Soluble fraction (wt%)[a]	Insoluble fraction (wt%)[a]	T_m (°C)[b]	FWHM(°C)[b]
1	64.3	100.0	-	217.4	8.7
2	14.3	100.0	-	195.6	22.1
3	9.6	88.5	11.5	188.2	16.1
4	9	5.0	95	162.8	20.6
5	1	1.8	98.2	78.9	47.8

[a]-determined using TFAA functionalization method.

[b]-determined using DSC results.

Table S13. Fractions of TFFA soluble and insoluble total solids after catalyzed reaction (2.3Ru/ZrO2), and corresponding melting point temperature, and FWHM determined by DSC for reaction time 1h and polymer-to-mass ratio 10:1.

Time (h)	Total solids yield (wt%)	Soluble fraction(wt%)[a]	insoluble fraction (wt%)[a]	T_m (°C)[b]	FWHM(°C)[b]
1	46	100	-	210.8	6

[a]-determined using TFAA functionalization method.

[b]-determined using DSC results

Here when the amount of catalyst was doubled, the amount of insoluble solids obtained was the same as the amount of catalyst in the sample. Hence all the solids were considered soluble. Note that total solids obtained are mixed with the catalyst. For instance, after 2 h of experiments, solid obtained had 25.8 wt.% of catalyst and the insoluble content in TFAA corresponded to 25.9 wt.%. After 3 h, insoluble solids became dominant with catalyst hence the TFAA solubility was no longer analyzed.

Table S14. DSC analysis of residual solid obtained as per experiment protocol in Figure 6.

**Guage pressure**	**T_m (°C)**	**FWHM(°C)**
Atm	215.2	11.6
10	210.2	4.5
20	207.4	6.3
30	195.7	22.1
36	198.8	8.2

Table S15. DSC analysis of residual solid obtained as per experiment protocol in Figure S12.

T_m (°C)	FWHM(°C)
**217.21**	**15.7**

Table S16. DSC Analysis of residual solid obtained as per experimental protocol in Figure 7a.

Wt% relative to nylon-6	T_m (°C)	FWHM (°C)
10	179.9	18.2
20	200.8	14.2
50	199.5	13.9

Table S17. DSC analysis of residual solid obtained as per experimental protocol in Figure 7b.

Wt% relative to nylon-6	T_m (°C)	FWHM(°C)
1	207.6	5
5	208.8	10.3
10	210.7	10.1

5. Catalyst reusability test.

a) TGA

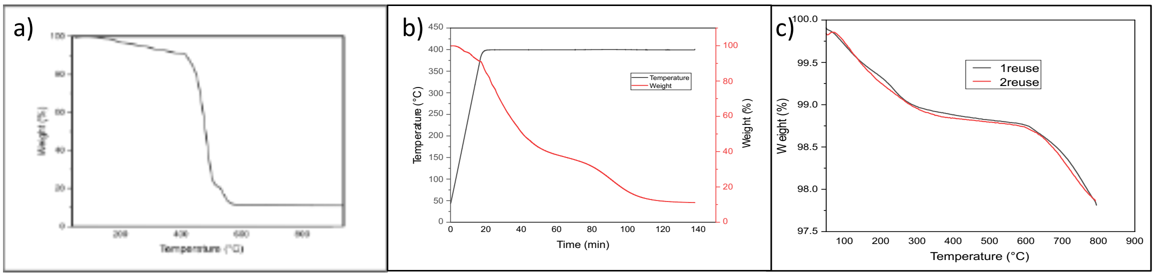

Figure S 12: TGA of residual solids along with catalyst collected after the experiment. Reaction conditions: 57g hexadecane solvent, 3 g of nylon-6, polymer-to-catalyst mass ratio of 20, gas pressure 30 bar H_2, and 2 h reaction time; a) at dynamic conditions from 40-900° C, b) isothermal condition at 400 °C, c) after regenerative calcination at 400 °C prior to first reuse (black) and second reuse (red).

b) N_2-Pysisorption

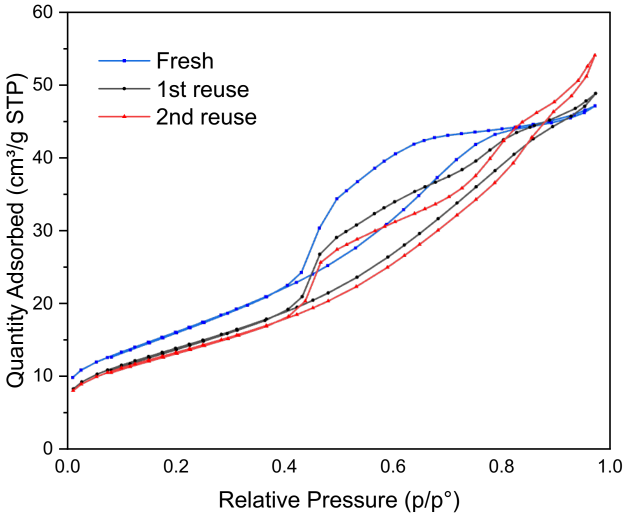

Figure S 13:N_2 physisorption isotherm of 2.3Ru/ZrO2 fresh catalyst (blue), regenerated catalyst before first reuse (black), and regenerated catalyst before second reuse (red).

References

(1) Biagini, E.; Gattiglia, E.; Pedemonte, E.; Russo, S. On the trifluoracetylation reaction of polyamides and polyurethanes. *Die Makromolekulare Chemie* **1983**, *184* (6), 1213-1222. DOI: 10.1002/macp.1983.021840609 (acccessed 2024-01-23T12:15:47).

(2) Millot, C.; Fillot, L.-A.; Lame, O.; Sotta, P.; Seguela, R. Assessment of polyamide-6 crystallinity by DSC. *Journal of Thermal Analysis and Calorimetry* **2015**, *122* (1), 307-314. DOI: 10.1007/s10973-015-4670-5 (acccessed 2024-04-01T21:00:40).
